# miRNA Expression Characterizes Histological Subtypes and Metastasis in Penile Squamous Cell Carcinoma

**DOI:** 10.3390/cancers13061480

**Published:** 2021-03-23

**Authors:** Hiresh Ayoubian, Joana Heinzelmann, Sebastian Hölters, Oybek Khalmurzaev, Alexey Pryalukhin, Philine Loertzer, Julia Heinzelbecker, Stefan Lohse, Carol Geppert, Hagen Loertzer, Heiko Wunderlich, Rainer M. Bohle, Michael Stöckle, Vsevolod Borisovich Matveev, Arndt Hartmann, Kerstin Junker

**Affiliations:** 1 Department of Urology and Paediatric Urology, Saarland University, 66421 Homburg, Germany; Hiresh.Ayoubian@uks.eu (H.A.); joana.heinzelmann@uk-halle.de (J.H.); sebastian.hoelters@t-online.de (S.H.); oyka0201@mail.ru (O.K.); ploertzer@westpfalz-klinikum.de (P.L.); Julia.heinzelbecker@uks.eu (J.H.); Michael.Stoeckle@uks.eu (M.S.); 2 Department of Ophthalmology, Martin-Luther-University Halle-Wittenberg, 06120 Halle/Saale, Germany; 3 Department of Urology, Federal State Budgetary Institution “N.N. Blokhin National Medical Research Center of Oncology” of the Ministry of Health of the Russian Federation, 115478 Moscow, Russia; vsevolodmatveev@mail.ru; 4 Institute of Pathology, Saarland University Medical Centre, 66421 Homburg, Germany; pryaluckin@mail.ru (A.P.); Rainer.Bohle@uks.eu (R.M.B.); 5 Westpfalz-Klinikum, Clinic of Urology and Paediatric Urology, 67655 Kaiserslautern, Germany; uro@westpfalz-klinikum.de; 6 Institute of Virology, Saarland University, 66421 Homburg, Germany; stefan.lohse@uni-saarland.de; 7 Institute of Pathology, University Erlangen-Nuremberg, 91054 Erlangen, Germany; carol.geppert@uk-erlangen.de (C.G.); arndt.hartmann@uk-erlangen.de (A.H.); 8 St. Georg Klinikum, Clinic of Urology and Paediatric Urology, 99817 Eisenach, Germany; wunderlich.heiko@stgeorgklinikum.de

**Keywords:** PSCC, miRNA, HPV, microarray

## Abstract

**Simple Summary:**

Penile squamous cell carcinoma (PSCC) is the most common type of penile cancer (PeCa) and is associated with human papillomavirus (HPV) in about 50% of cases. It is of high clinical impact to identify patients who are at high risk of metastasis and are likely to benefit from adjuvant therapies. Today, valid prognostic biomarkers are scarce in penile cancer. In the present study, we attempted to identify miRNAs involved in tumor development and metastasis in distinct histological subtypes with or without HPV infection in PSCC patients. We confirmed that specific miRNAs could serve as potential diagnostic and prognostic markers in single PSCC subtypes and are associated with HPV infection.

**Abstract:**

Although microRNAs are described as promising biomarkers in many tumor types, little is known about their role in PSCC. Thus, we attempted to identify miRNAs involved in tumor development and metastasis in distinct histological subtypes considering the impact of HPV infection. In a first step, microarray analyses were performed on RNA from formalin-fixed, paraffin-embedded tumor (22), and normal (8) tissue samples. Microarray data were validated for selected miRNAs by qRT-PCR on an enlarged cohort, including 27 tumor and 18 normal tissues. We found 876 significantly differentially expressed miRNAs (*p* ≤ 0.01) between HPV-positive and HPV-negative tumor samples by microarray analysis. Although no significant differences were detected between normal and tumor tissue in the whole cohort, specific expression patterns occurred in distinct histological subtypes, such as HPV-negative usual PSCC (95 differentially expressed miRNAs, *p* ≤ 0.05) and HPV-positive basaloid/warty subtypes (247 differentially expressed miRNAs, *p* ≤ 0.05). Selected miRNAs were confirmed by qRT-PCR. Furthermore, microarray data revealed 118 miRNAs (*p* ≤ 0.01) that were significantly differentially expressed in metastatic versus non-metastatic usual PSCC. The lower expression levels for miR-137 and miR-328-3p in metastatic usual PSCC were validated by qRT-PCR. The results of this study confirmed that specific miRNAs could serve as potential diagnostic and prognostic markers in single PSCC subtypes and are associated with HPV-dependent pathways.

## 1. Introduction

The incidence of penile cancer (PeCa) is low at 0.3 to 1.0 per 100,000 in Western Europe and the United States. The most common PeCa type is penile squamous cell carcinoma (PSCC) [1,2,3]. A recent systematic review and meta-analysis of the published literature found that human papillomavirus (HPV) DNA is detectable in 50.8% of penile cancer cases, of which HPV-16 is the most prevalent viral subtype, accounting for 63% of the HPV-attributable cases, followed by HPV-6 (8.1%), HPV-18 (6.9%), and, to a lesser extent, HPV-11, HPV-45, HPV-33, HPV-52, and HPV-31, among others [4]. As such, the 2016 WHO classification categorizes PSCC into HPV-related (such as basaloid, warty, and warty–basaloid) and non-HPV-related subtypes, which include the usual histological subtype as the most common [5,6]. Previous studies have explained two different mechanisms for oncogenesis in PSCC. First, the HPV-independent mechanism is linked to p53 mutation and shows nuclear p53 expression in atypical basal keratinocytes without P16INK4a expression [7,8,9]. P16INK4a downregulation through overexpression of the *BMI-1* polycomb gene and inactivation of P16INK4a through DNA methylation has also been described in PSCC [10,11]. Second, the HPV-dependent mechanism involves E6 and E7 viral oncoproteins binding to *p53* and retinoblastoma gene protein Rb. These host regulatory proteins disrupt DNA repair, growth arrest, apoptosis via E6 [12], and inactivation of hypo-phosphorylated Rb protein via E7, resulting in nuclear accumulation of cyclin-dependent kinase inhibitor P16INK4a [10,13,14,15].

In contrast to the well-known role of HPV in tumor development, the impact of HPV infection on the prognosis of PSCC remains unclear and is still under discussion [16,17,18].

It is of high clinical impact to identify patients who are at a high risk of metastasis and are likely to benefit from invasive therapies such as early inguinal lymph node dissection or adjuvant systemic chemotherapy. We and others proved regional lymph node involvement and lymph vessel invasion as independent prognostic factors in PSCC [19,20,21]. However, these parameters are not sufficient for individual therapy selection to improve treatment in high-risk patients and to prevent adverse effects in low-risk patients. Therefore, prognostic biomarkers are urgently needed.

miRNAs are conserved, short, single-stranded, non-coding RNAs of 19 to 25 nucleotides involved in post-transcriptional regulation of target genes by binding to the 3′ untranslated regions (3′UTRs) of the messenger RNA (mRNA) [22,23]. It has been shown that miRNAs play a crucial role in a broad range of biological processes, such as the proliferation, metabolism, apoptosis, and metastasis of tumors [24,25,26]. Changes in miRNA expression have been reported in a variety of human tumors and can potentially be employed as clinical biomarkers of diagnosis, prognosis, metastasis, and therapy resistance [27,28]. It was previously revealed that HPV infection via viral oncoproteins E6 and E7 altered the expression of cellular oncogenic and tumor suppressor miRNAs by modulating the *c-Myc*, *p53*, and *E2F* genes [29,30,31]. While it can be concluded that HPV infection has an effective impact on many biological processes, particularly through its interaction with the miRNA network, the impact of HPV infection on miRNA expression in PSCC remains unclear [16,17,18]. Furthermore, little is known about the role of miRNAs in tumor development and progression in this tumor type in general, and even less in histological subtypes. Therefore, miRNA expression analyses were performed in the present study to identify deregulated miRNAs associated with tumorigenesis and metastasis in distinct histological subtypes of PSCC considering HPV status.

## 2. Materials and Methods

### 2.1. Sample Collection and Patient Data

The patient samples were obtained from urology departments in Germany and Russia as described in our previous cohort study [19] (Table 1 and Table S1). Thirty samples, including 22 tumor tissue and 8 normal tissue samples, were used for screening by microarray analysis. For validation by PCR, 45 samples (27 tumor and 18 normal tissue samples) were analyzed, including those from the screening cohort. The hematoxylin and eosin (H&E)-stained tissue sections were assessed by two pathologists to confirm histologic subtypes as previously described [19]. Written informed consent was obtained from patients in most cases. For those individuals from whom we could not obtain informed consent, the data were analyzed anonymously. A regional ethics committee approved this investigation (Saarland ethical committee votes: 42/17, 220/19).

### 2.2. DNA and RNA Isolation

Total DNA and RNA (including small RNA) were isolated from formalin-fixed, paraffin-embedded (FFPE) tissue sections, prepared by punches from defined tumor-affected or normal areas, using AllPrep^®^ DNA/RNA FFPE Kits (Qiagen, Hilden Germany) according to the manufacturer’s instructions. Detection of HPV infection was performed by HPV polymerase chain reaction (PCR) for GP5+/6+ and Glyceraldehyde 3-phosphate dehydrogenase (GAPDH) primers using the LightCycler^®^ 1.5 instrument (Roche Diagnostics GmbH) as previously described [19,32]. The concentration and purity of the RNA was assessed using the ratio of absorbance levels at 230, 260, and 280 nm, determined using a NanoDrop™ ND-1000 Spectrophotometer (ThermoFisher Scientific, Dreieich, Germany).

### 2.3. miRNA Microarray and Data Analysis

MicroRNA labelling and hybridization were performed using the miRNA Complete Labelling and Hybridization Kit (Agilent, Waldbronn, Germany) according to the manufacturer’s instructions. Briefly, 100 ng of isolated RNA from PSCC and normal tissue was dephosphorylated, labelled with pCp-Cy3, and hybridized to the Agilent human miRNA microarray. Microarray data analysis was performed using Qlucore Omics Explorer 3.5 (Qlucore AB, Lund, Sweden). In brief, microarrays were scanned using the Agilent Microarray Scanner, and expression values were extracted using the Agilent Feature Extraction (FE) software. Unpaired Student t-tests were performed using Qlucore Omics Explorer. The differential expression of miRNA between groups was considered statistically significant when *p*-values were less than 0.05, false discovery rates (*q*) were less than 0.3, and a 2-fold change in miRNA expression was detected. The total gene signal was normalized to the 75th percentile of signal intensity. Unsupervised hierarchical clustering and principal component analysis (PCA) were also conducted using Qlucore Omics Explorer.

### 2.4. Real-Time RT-PCR Quantification of miRNAs

To validate the miRNA microarray data, reverse transcription PCR (qRT-PCR) was performed using TaqMan^®^ MicroRNA Reverse Transcription Kits or TaqMan^®^ Advanced miRNA cDNA Synthesis Kits, followed by a polymerase chain reaction (PCR) step using specific TaqMan^®^ miRNA primers and TaqMan^®^ Fast Advanced Master Mix, according to the manufacturer’s protocol. PCRs were run in triplicate using LightCycler^®^ 480 (Roche diagnostics Deutschland GmbH, Mannheim, Germany). miR-191-5p (ThermoFisher) was used as an endogenous control to normalize miRNA input in the real-time PCR (qRT-PCR) assay. The relative quantitative method of 2^−ΔΔCT^ was used to measure changes in selected miRNAs. The ΔCt value was calculated by subtracting the Ct values of miR-191-5p from the Ct values of the miRNA of interest. Deregulation of miRNA was considered validated if there was at least a 2-fold expression difference between each group and the deregulation was in the same direction as in the microarray data. Ct values above 35 were considered as not expressing miRNA. The nonparametric Mann–Whitney U test was used to analyze the qRT-PCR data (IBM^®^ SPSS^®^ Statistics version 25). A *p*-value of less than 0.05 was considered a statistically significant threshold for upregulated or downregulated miRNA.

## 3. Results

### 3.1. Microarray Analysis

**Figure 1:**
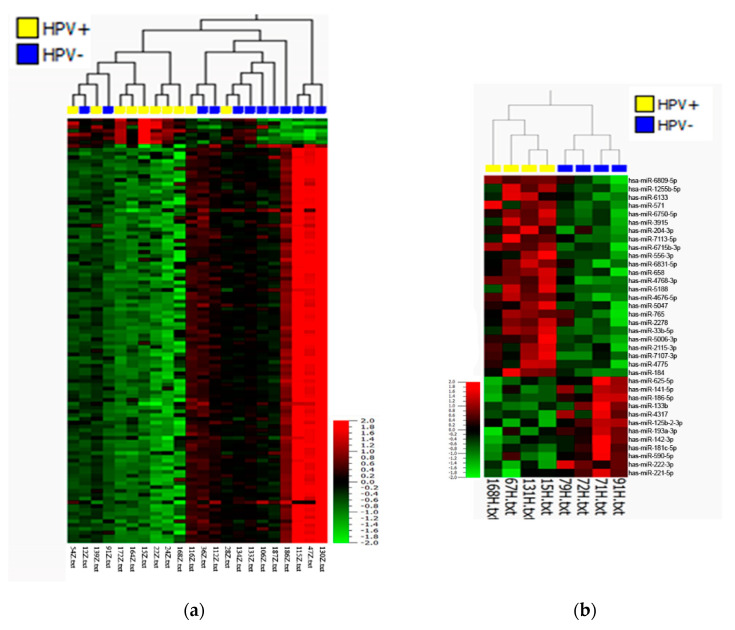
Heat map after unsupervised hierarchical clustering of differentially expressed miRNAs in HPV-positive versus HPV-negative (**a**) PSCC tumor tissues (*p* ≤ 0.01; *q* ≤ 0.027; fold change ≥ 2) and (**b**) normal tissues (*p* ≤ 0.05; *q* ≤ 0.37; fold change ≥ 2).

**Figure 2:**
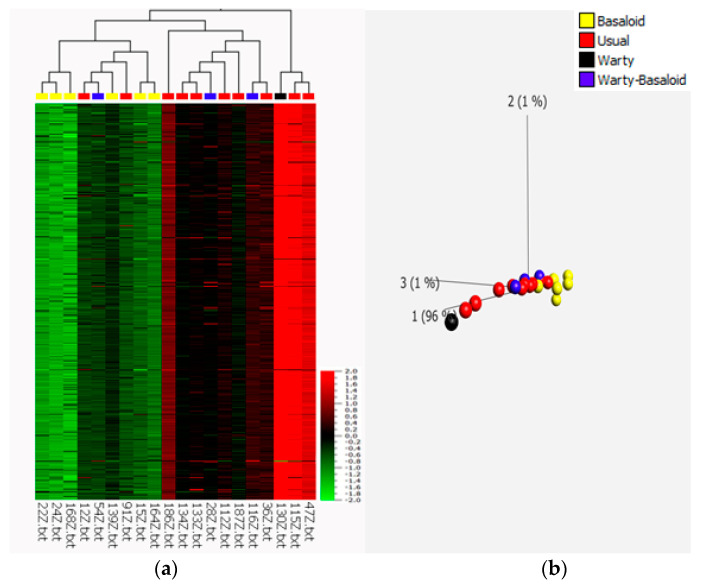
Multiple group comparison of differentially expressed miRNAs in basaloid, warty–basaloid, warty, and usual histological subtypes using the F-test (*q* ≤ 0.01). (**a**) Heatmap; (**b**) principal component analysis plot.

To determine the possible association between HPV infection and miRNA expression and to test whether miRNAs could serve as diagnostic or prognostic markers, 12 HPV-negative and 10 HPV-positive tumor samples of different histological subtypes, as well as 8 non-tumor tissue samples related to both HPV-positive and HPV-negative tumors, were subjected to miRNA microarray analysis. The comparison of HPV-positive and HPV-negative cases revealed significant differences in miRNA expression profiles (Figure 1a). In total, 876 miRNAs showed significantly different expression between HPV-positive and HPV-negative tumors (*p* ≤ 0.01, *q* ≤ 0.027), including 868 downregulated and 8 upregulated miRNAs in HPV-positive PSCC (Table S2). However, we could not find statistically significant differences in miRNA expression profiles between HPV-related and non-HPV-related histological subtypes (Figure S1) (*q* ≥ 0.81). There was also a statistically significant difference between non-tumor tissues based on HPV status (*p* ≤ 0.05, *q* ≤ 0.37), resulting in the upregulation of 24 miRNAs and the downregulation of 12 miRNAs in HPV-positive cases (Figure 1b). Surprisingly, we did not find significant differences in miRNA expression between tumor and normal samples in general (*q* ≥ 0.99) (Figure S2). From PCA and hierarchical clustering of the miRNA expression profiles of usual, warty, basaloid, and warty–basaloid PSCC subtypes, we found distinct miRNA profiles of basaloid tumors, and we found the usual subtype to be closer to the warty and warty–basaloid subtypes (F ≤ 0.01; Figure 2).

**Figure 3:**
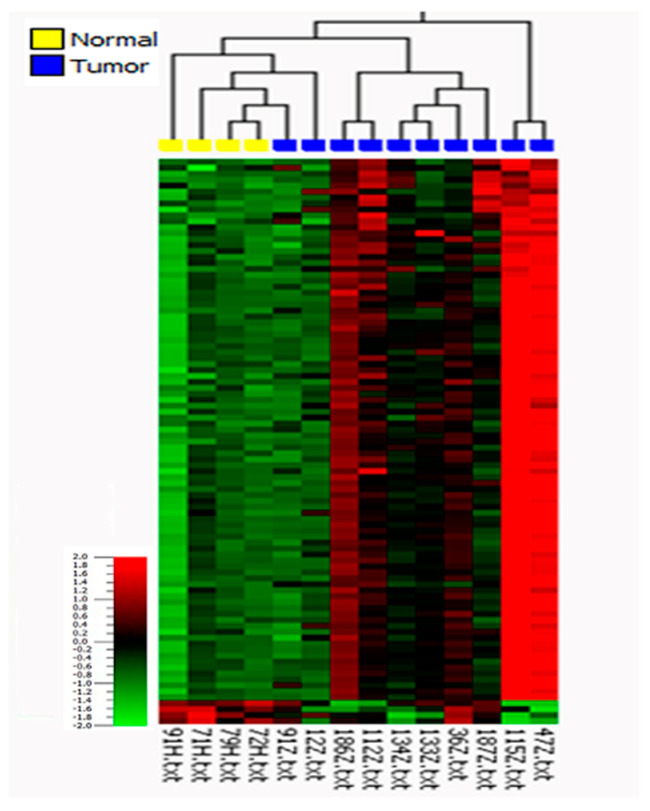
Heat map after unsupervised hierarchical clustering of differentially expressed miRNAs in usual (HPV-negative) tumors versus related normal tissue samples (*p* ≤ 0.05; *q* ≤ 0.23; fold change ≥ 2).

**Figure 4:**
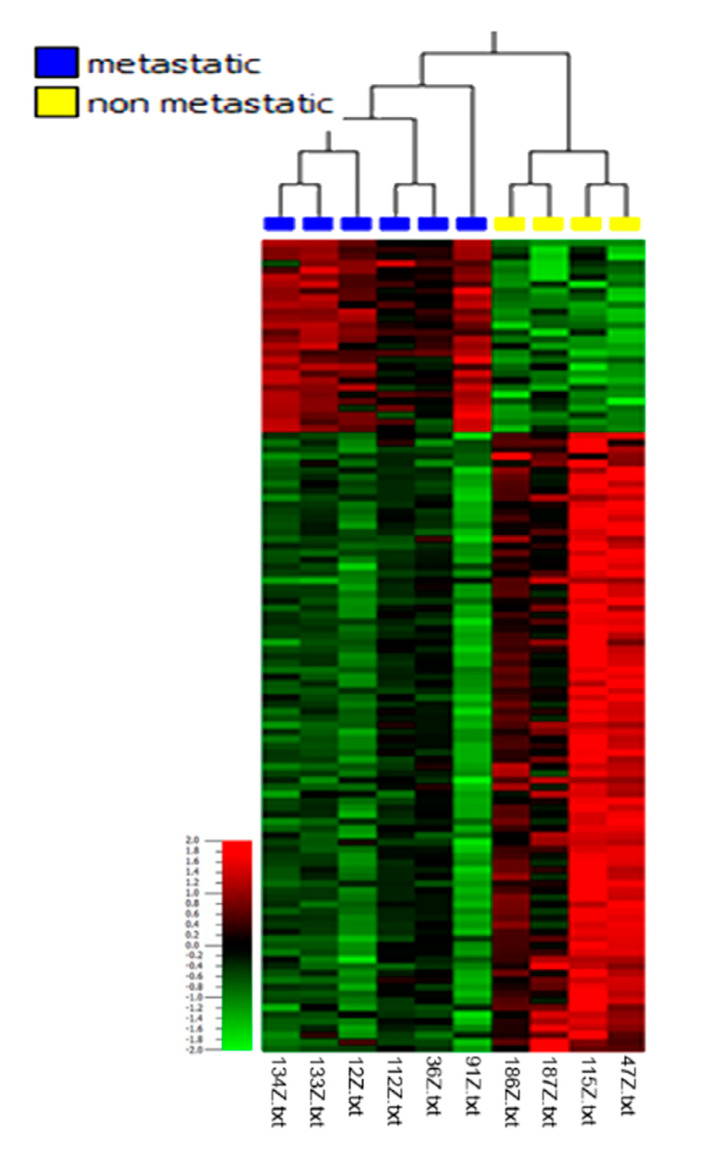
Heat map after unsupervised hierarchical clustering of differentially expressed miRNAs in metastatic versus non-metastatic (HPV-negative) usual PSCC (*p* ≤ 0.01; *q* ≤ 0.08; fold change ≥ 2).

Based on these differences, we further investigated miRNA expression in the context of histological subtypes considering HPV status. A comparison of the profiles of the HPV-negative usual subtype and normal tissues revealed 95 differentially expressed miRNAs, including 91 that were upregulated and 4 that were downregulated in tumor tissue with a 2-fold change (*p* ≤ 0.05, *q* ≤ 0.23; Figure 3 and Table S3). In this comparison, miR-4512 had the highest expression, with a 5.1-fold increase, and miR-99a-5p had the lowest expression, with an 8-fold decrease, relative to normal tissue. To identify deregulated miRNAs involved in metastasis within this subgroup, the miRNA expression profiles in metastatic versus non-metastatic tissues were then analyzed (Figure 4 and Table S4). They were found to be statistically significantly different (*p* ≤ 0.01, *q* ≤ 0.08), with a total of 118 significantly deregulated miRNAs, of which 28 were upregulated and 90 were downregulated in metastatic tumors. Here, miR-4498 had the highest expression, with a 5.4-fold increase, whereas miR-137 (with a 3.7-fold decrease) and miR-301b (with a 4-fold decrease) had the lowest expression in usual metastatic tumors.

**Figure 5:**
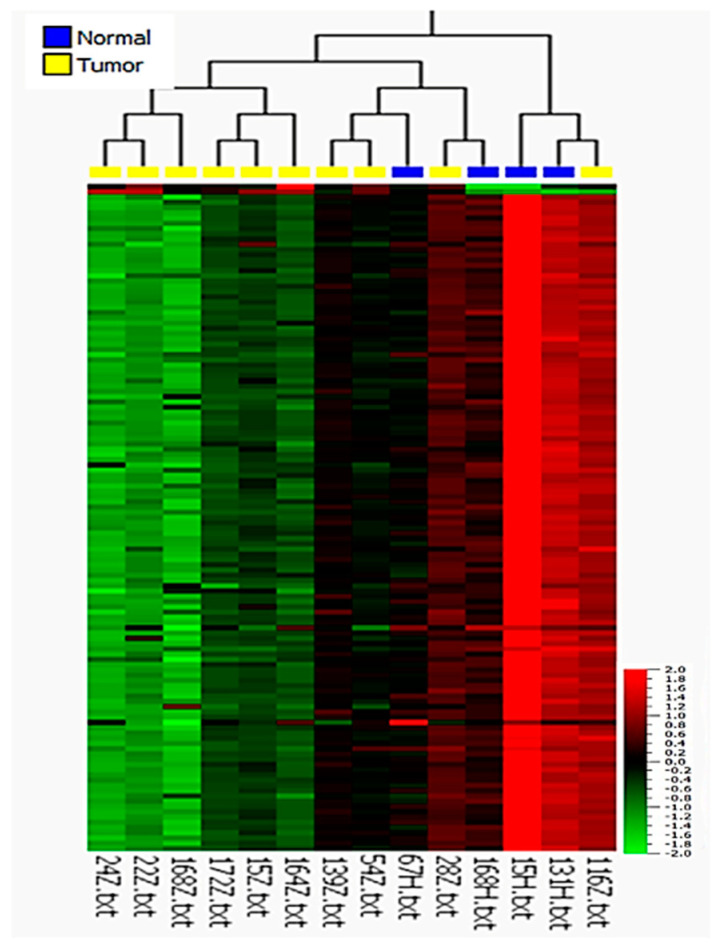
Heat map after unsupervised hierarchical clustering of differentially expressed miRNAs in basaloid, warty, and warty–basaloid (HPV-positive) PSCC versus related normal tissue samples (*p* ≤ 0.05; *q* ≤ 0.17; fold change ≥ 2).

Due to the low frequency of basaloid tumors and the histological similarities between warty, basaloid, and warty–basaloid tumors, these three PSCC subtypes were combined into a single group. A comparison of this HPV-positive group to the related normal tissues revealed 247 miRNAs that were differently expressed (*p* ≤ 0.05, *q* ≤ 0.17). The majority of the miRNAs (*n* = 245) were downregulated, while only two were upregulated (Figure 5). Here, miR-181d-5p had the highest expression, with a 3.41-fold increase, whereas miR-211-5p had the lowest expression, with a 16.96-fold decrease in tumor tissue (Table S5). The analysis of microarray data did not reveal statistically significantly deregulated miRNAs between metastatic and non-metastatic tissues in these subtypes (*q* ≥ 0.79; Figure S3).

### 3.2. Validation of Differentially Expressed miRNAs by qRT-PCR

**Figure 6:**
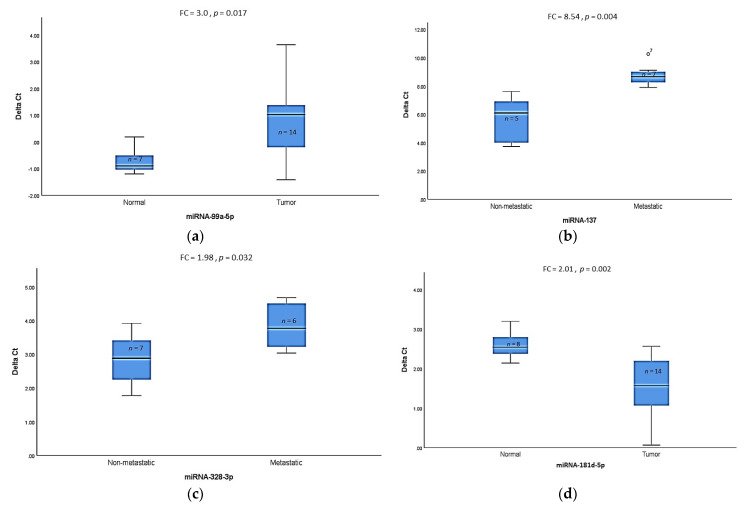
Validation of differentially expressed miRNAs in different PSCC histological subtypes as determined by TaqMan^®^ RT-qPCR (*p* ≤ 0.05). (**a**) Mean ΔCt values of (HPV-negative) usual PSCC and normal tissue samples; (**b**,**c**) mean ΔCt values of metastatic versus non-metastatic usual (HPV-negative) tumors; (**d**) mean ΔCt values of warty–basaloid (HPV-positive) PSCC versus normal tissue samples. Lower ΔCt implies higher expression level. Mann–Whitney U test was used to determine *p*-values, FC: Fold change.

Based on the results of the microarray analysis (*p*- and *q*-values and fold change) and the potential biological functions in the context of tumorigenesis and metastasis, we selected eight miRNAs for validation using TaqMan^®^ RT-qPCR from an enlarged cohort, including samples used for the microarray analyses. We focused on analysis in specific subtypes as described before. The significantly lower expression level for miR-99a-5p (*p* = 0.017) in the HPV-negative usual subtype compared to normal tissue samples was validated (Figure 6a). No statistically significant differences were found for either miR-125b-2-3p or miR-105-5p in this subgroup comparison (Figure S4), despite significant differences in microarray analysis. Furthermore, significantly lower expression levels for miR-137 (*p* = 0.004) and miR-328-3p (*p* = 0.032) in metastatic usual subtypes compared to non-metastatic tumors were also confirmed (Figure 6b,c). No significant difference was found for miR-509-5p in this subgroup comparison (Figure S4). Finally, we validated the higher expression of miR-181d-5p (*p* = 0.002) (Figure 6d), and no or very low expression of miR-211-5p was found in the HPV-positive basaloid, warty, and warty–basaloid subtypes compared to normal tissue (Table 2). Due to the non-significant results of microarray analysis when comparing the metastatic and non-metastatic tumors, we did not perform further qRT-PCR experiments.

## 4. Discussion

Currently, no biomarkers are available for the differential diagnosis, prognostic evaluation, or therapy response prediction of PSCC. However, this is a clinical need since individual prognostic evaluation and, therefore, treatment decisions concerning prevention of or invasive therapy for metastatic dissemination are still a challenge in this rare but often aggressive disease.

HPV infection is one of the most common risk factors in PSCC [1]. The prognostic role of HPV, however, is still debated [19,33,34,35,36]. The current WHO classification of PSCC is now based on clinicopathologic uniqueness and HPV status, and it assigns histological subtypes to HPV-related and non-HPV-related PSCC [5]. It should be considered that there is great diversity among histopathological subtypes in the two major groups concerning aggressiveness and outcome. Whereas verrucous and pseudohyperplastic PSCC are well differentiated and have an excellent prognosis, pseudoglandular and sarcomatoid PSCC are aggressive tumors with poor outcomes, and all are non-HPV-related PSCC. Usual PSCC represents the most frequent non-HPV-related subtype with heterogeneous outcome. In HPV-related PSCC, the basaloid subtype represents a poorly differentiated tumor type with a mainly worse disease course. Therefore, individual histopathological subtypes should be considered in clinical and molecular studies in addition to the HPV-status.

In the present study, we investigated miRNA expression profiles considering histopathological subtypes and HPV status. Microarray analysis showed that 876 miRNAs are differentially expressed at statistically significant levels in HPV-positive relative to HPV-negative PSCC. This suggests that HPV infection may indeed affect the expression of miRNAs in PSCC, resulting in distinct molecular pathways, as shown previously [36,37,38]. Interestingly, a positive HPV status in normal tissue was also associated with changes in miRNA expression. This could explain why statistically significant differences in miRNA expression profiles between tumor and normal tissues in general were not identified. In contrast to the real HPV status, a comparison of HPV-related (subtypes with predominant but not exclusive HPV infection based on histological subtypes) and non-HPV-related (subtypes with predominantly negative HPV status with only some HPV infection based on histological subtypes) PSCC did not reveal statistically significant changes in miRNA expression as defined by the current WHO classification system [5]. In concordance with published data, we found that 23% of HPV-negative PSCC are classified as HPV-related based on histological subtype. These data suggest that real HPV infection, not classification in HPV-related groups, is the relevant biological event associated with miRNA expression.

As our previous findings support the assertion that histological subtypes are more relevant than HPV status for prognosis [19], miRNA expression in different histological subtypes was further analyzed. Since PSCC is a rare tumor, little is known about miRNA alterations in distinct histological PSCC subtypes. The results of the present study confirm that histological subtypes are characterized by distinct miRNA expression profiles, especially between basaloid and usual PSCC. Therefore, the miRNA expression in these two main histological subtypes was further separately investigated. In contrast to the whole cohort, statistically significant differences in miRNA expression were identified between normal and tumor tissues in the HPV-negative usual and HPV-positive basaloid, warty, and warty–basaloid PSCC subtypes. To validate the microarray data, quantification via qRT-PCR was performed for selected miRNAs. We confirmed the lower expression of miR-99a-5p in tumor tissues compared to normal tissues in the usual PSCC subtype, supporting data on the tumor-suppressive role of this miRNA in other tumor types [39,40,41].

In the miRNA expression profile comparison of the warty, basaloid, and warty–basaloid group versus normal tissue, miR-211-5p and miR-181d-5p were the most downregulated and upregulated miRNAs, respectively. Interestingly, miR-211-5p expression in this group was not detected at all in tumor tissues based on the cut-off criteria in qRT-PCR analysis. These results are in line with the microarray data, which found a 16-fold downregulation in miR-211-5p in this group compared to normal samples. These data are consistent with a previous investigation of small RNA sequencing that found a 300-fold lower expression of miR-211-5p in cancerous penile tissues relative to adjacent non-cancerous tissues [42]. miR-211-5p has been reported to play a crucial role as a tumor suppressor in various cancer types [43,44,45,46]

The results of the qRT-PCR analysis also confirmed the overexpression of miR-181d-5p. The upregulation of miR-181d-5p has been found to induce hepatocarcinogenesis [47]. This miRNA packed in exosomes secreted by cancer associated fibroblasts enhances the epithelial–mesenchymal transition (EMT) and aggressiveness of breast cancer by targeting caudal-related homeobox 2 (CDX2) [48]. CDX2 loss or reduced expression has been associated with advanced tumor stage, including metastasis, as well as poor prognosis in colorectal cancer [49,50]. It is therefore possible that miR-181d-5p overexpression directly or indirectly reduces the expression of the CDX2 protein in PSCC, enforcing tumorigenesis.

We were then interested in finding putative prognostic miRNAs. We could not detect miRNA expression differences in association with metastasis in the whole cohort (data not shown). Therefore, we analyzed altered miRNAs possibly involved in metastasis by comparing metastatic and non-metastatic HPV-negative usual tumors. We found a clear pattern that characterized metastatic primary tumors by microarray analysis. The lower expression of miR-137 and miR-328-3p was confirmed by qRT-PCR. It has been shown that miR-137, as a tumor suppressor, is downregulated in many cancer types [51,52,53]. miR-137 overexpression inhibits tumor growth and increases chemosensitivity to paclitaxel and cisplatin in lung cancer cell lines [54]. In this regard, it would be interesting to investigate the putative role of this miRNA for chemotherapy response in PSCC, too. Downregulation of miR-328-3p has been linked to its role as a tumor metastasis suppressor by targeting matrix metalloproteases [55,56,57]. Zhang et al. also showed downregulation of miR-328-3p in penile cancer tissues relative to adjacent non-cancerous tissues by Next-Generation Small RNA Sequencing [42]. MiRmiR-328-3p inhibits metastasis in colorectal cancer via inactivation of the PI3K/Akt signaling pathway [58] and reduces invasion as well as EMT in liver cancer via by targeting endoplasmatic reticulum metallo protease 1 (*ERMP1*) to inhibit AKT phosphorylation [59].

We also investigated differential miRNA expression between metastatic and non-metastatic tumors in combined basaloid, warty, and warty–basaloid subtypes. However, the heterogeneity in this group was greatly elevated, as reflected by the high false discovery rate (*q* ≥ 0.79), perhaps due to the small sample size and heterogeneity. Therefore, the results were not sufficient for validation using qRT-PCR in this study, and this will be explored in the future with a larger sample cohort. Our data confirmed that distinct miRNAs could serve as prognostic biomarkers in primary tumor tissues to predict metastasis and could therefore support decision-making for local lymph node treatment, as well as adjuvant therapy.

It is accepted that the tumor microenvironment plays an important role in tumor development and progression. In this regard, immune checkpoints are of high interest not only as prognostic markers, but also as therapeutic targets. High programmed death-ligand 1 (PD-L1) expression is associated with poor prognosis in a variety of solid tumors [60,61,62,63]. Forty-eight percent of PSCC cases are PD-L1-positive; these are primarily associated with HPV-negative subtypes and poor survival [64]. PD-L1 expression is associated with lymph node metastasis in PSCC patients [64,65]. The correlation between miRNA expression—including that of miR-138-5p, miR-15a, miR-15b, miR-16, miR-34a, miR-142-5p, and miR-200—and PD-L1 is well known [60,66,67,68,69]. We found by microarray analysis that miR-138-5p was upregulated in usual PSCC. miR-138-5p is a tumor suppressor that targets PD-L1 [70,71,72]. Therefore, it would be interesting to investigate the potential role of miR-138-5p as a biomarker for therapy selection using checkpoint inhibitors in PSCC patients in the future.

In general, few data on miRNA expression in PSCC are published. Higher expression levels of miR-223-3p, miR-107, and miR-21 [36,42,73], and lower expression levels of miR-1, miR-101, and miR-204, have been reported as potential biomarkers in patients with PSCC [74]. However, we did not find significant expression differences for the mentioned miRNAs, which might be due to several reasons, including technical differences. We used punches from defined tumor and normal areas obtained from FFPE blocks to isolate RNA. miRNA expression was analyzed by microarrays instead of NGS with different bioinformatics tools. In addition, possibly more importantly, we investigated the altered miRNAs in respect to histological subtypes separately. Further studies should also include long noncoding RNAs as another important class of non-coding RNAs regulating the complex network of RNA translation.

## 5. Conclusions

This is the first study, to our knowledge, investigating miRNA expression in PSCC considering histological subtypes, HPV infection, and metastatic status in a single study. The identified miRNAs could serve as potential diagnostic and prognostic markers for single PSCC subtypes. Our data support the assertion that PSCC subtypes are characterized by distinct molecular alterations and should therefore be investigated separately, as already shown by clinical data [19]. It seems that HPV infection induces distinct miRNA expression in PSCC. The influence and degree of altered miRNAs associated with HPV infection in the different PSCC histological subtypes requires additional research in a larger cohort.

## Figures and Tables

**Table 1:** Summary of the clinical and histopathological data of the penile squamous cell carcinoma (PSCC) samples. n/a: Not available. In addition to lymph node metastases, visceral metastases were found in liver and lung. HPV: Human papillomavirus.

PATIENT CHARACTERISTICS		*N*	%
**HISTOLOGICAL SUBTYPE**	usual	18	51.4
	warty–basaloid	6	17.1
	basaloid	8	22.9
	warty	1	2.9
	clear cell	1	2.9
	verrucous	1	2.9
**PRIMARY TUMOR**	pT1a	8	22.9
	pT1b	4	11.4
	pT2	11	31.4
	pT3	10	28.6
	n/a	2	5.7
**REGIONAL LYMPH NODES**	N0	17	48.6
	N1	6	17.1
	N2	1	2.9
	N3	9	25.7
	n/a	2	5.7
**HISTOLOGIC GRADE**	G1	2	5.7
	G2	16	45.7
	G3	15	42.9
	n/a	2	5.7
**METASTASIS STATUS**	Non-metastatic	18	51.4
	Metastatic	17	48.6
**HPV STATUS**	HPV+	17	48.6
	HPV-	18	51.4

**Table 2:** Summary of miRNAs validated by microarray and RT-qPCR in different histological subtypes.

miRNAs	Fold Change in Microarray, *p*-Value	Fold Change in RT-qPCR, *p*-Value	HPV Status	Comparison	Histological Subtype
miR-99a-5p	−8.0 (*p* = 0.037)	−3.5 (*p* = 0.017)	HPV-negative	Tumor vs. Normal	Usual
miR-137	−3.7 (*p* = 0.004)	−8.54 (*p* = 0.004)	HPV-negative	Metastatic vs. Non-metastatic	
miR-328-3p	−2.7 (*p* = 0.007)	−1.98 (*p* = 0.032)	HPV-negative	Metastatic vs. Non-metastatic	
miR-181d-5p	+3.4 (*p* = 0.004)	+2.01 (*p* = 0.002)	HPV-positive	Tumor vs. Normal	Basaloid, Warty, and Warty–basaloid
miR-211-5p	−16.9 (*p* = 0.046)	Not defined	HPV-positive	Tumor vs. Normal	

## Data Availability

The data presented in this study are available in supplementary material tables and on request from the corresponding author.

## Supplementary Materials

The following data are available online at https://www.mdpi.com/2072-6694/13/6/1480/s1: Figure S1: Unsupervised hierarchical clustering of differentially expressed miRNAs in HPV-related versus non-HPV-related PSCC, Figure S2: Heat map after unsupervised hierarchical clustering of differentially expressed miRNAs in tumor versus normal tissues, Figure S3: Heat map after unsupervised hierarchical clustering of differentially expressed miRNAs in metastatic versus non- metastatic (HPV-positive) basaloid, warty, and warty basaloid histological subtype, Figure S4: miRNAs without significant differences as determined by TaqMan^®^ RT-qPCR, Table S1: Summary of clinical and histopathological data of the PSCC samples used for microarray analysis and qRT-PCR validation, Table S2: Significantly differentially expressed miRNAs in HPV-positive versus HPV-negative PSCC, Table S3: Significantly differentially expressed miRNAs in usual (HPV-negative) PSCC versus normal samples, Table S4: Significantly differentially expressed miRNAs in metastatic versus non-metastatic usual (HPV-negative) PSCC, Table S5: Significantly differentially expressed miRNAs in combined basaloid and warty basaloid (HPV-positive) PSCC versus normal tissue samples (HPV-positive).
