# Supplementary material for: miRNA Expression Characterizes Histological Subtypes and Metastasis in Penile Squamous Cell Carcinoma
Source: Cancers (Basel). 2021 Mar 23;13(6):1480. doi: 10.3390/cancers13061480 (PMC8004785; doi:10.3390/cancers13061480)
Supplement: Supplementary file 1 [file cancers-13-01480-s001.zip › cancers-1116103-supp/supplementary figures with legands.docx]

| 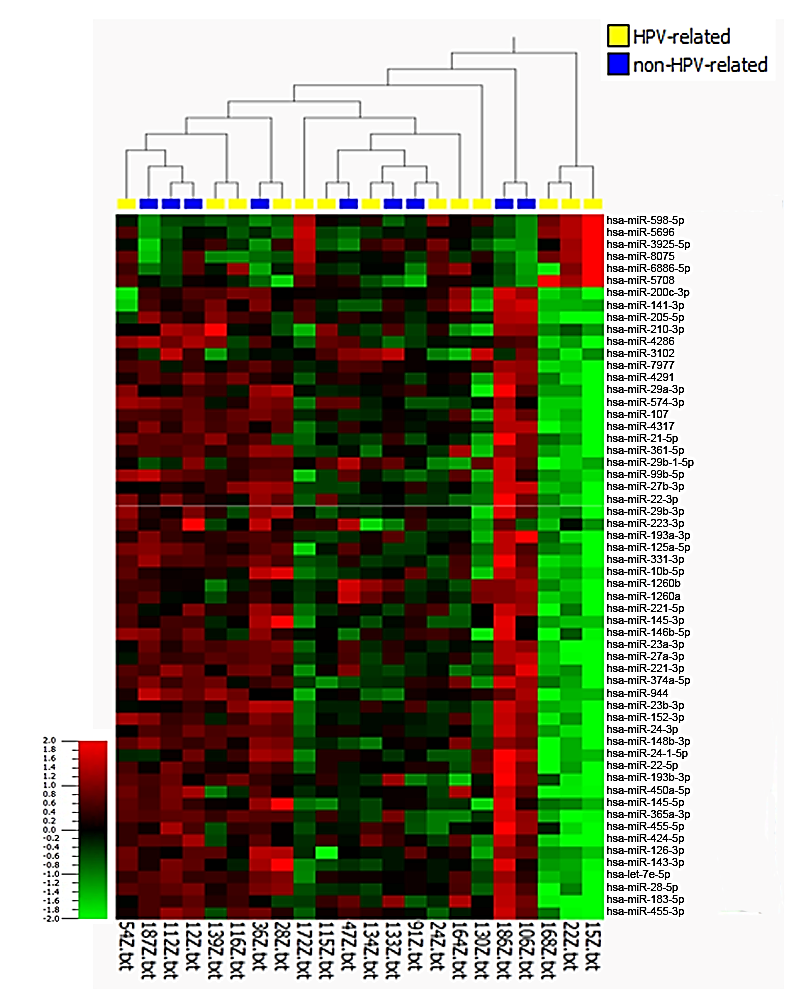 |
| --- |
| Figure S1. Unsupervised hierarchical clustering of differentially expressed miRNAs in HPV-related versus non-HPV-related PSCC (p ≤ 0.05; q ≥ 0.81; fold change ≥ 2). |

| 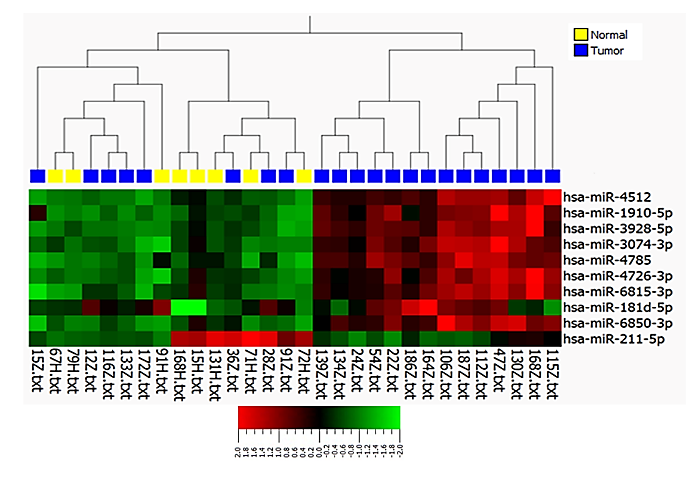 |
| --- |
| Figure S2. Heat map after unsupervised hierarchical clustering of differentially expressed miRNAs in tumor versus normal tissues (p ≤ 0.05; q ≥ 0.99; fold change ≥ 2). |

| 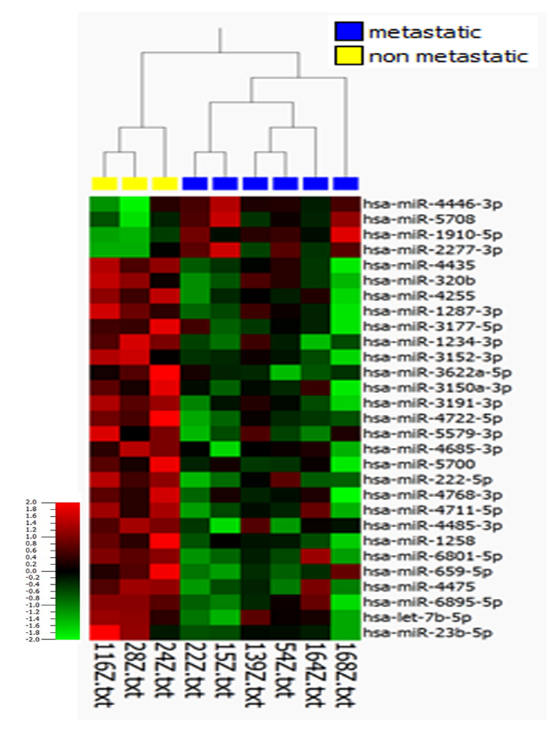 |
| --- |
| Figure S3. Heat map after unsupervised hierarchical clustering of differentially expressed miRNAs in metastatic versus non- metastatic (HPV-positive) basaloid, warty, and warty basaloid histological subtype (p ≤ 0.05; q ≥0.79; fold change ≥ 2). |

| 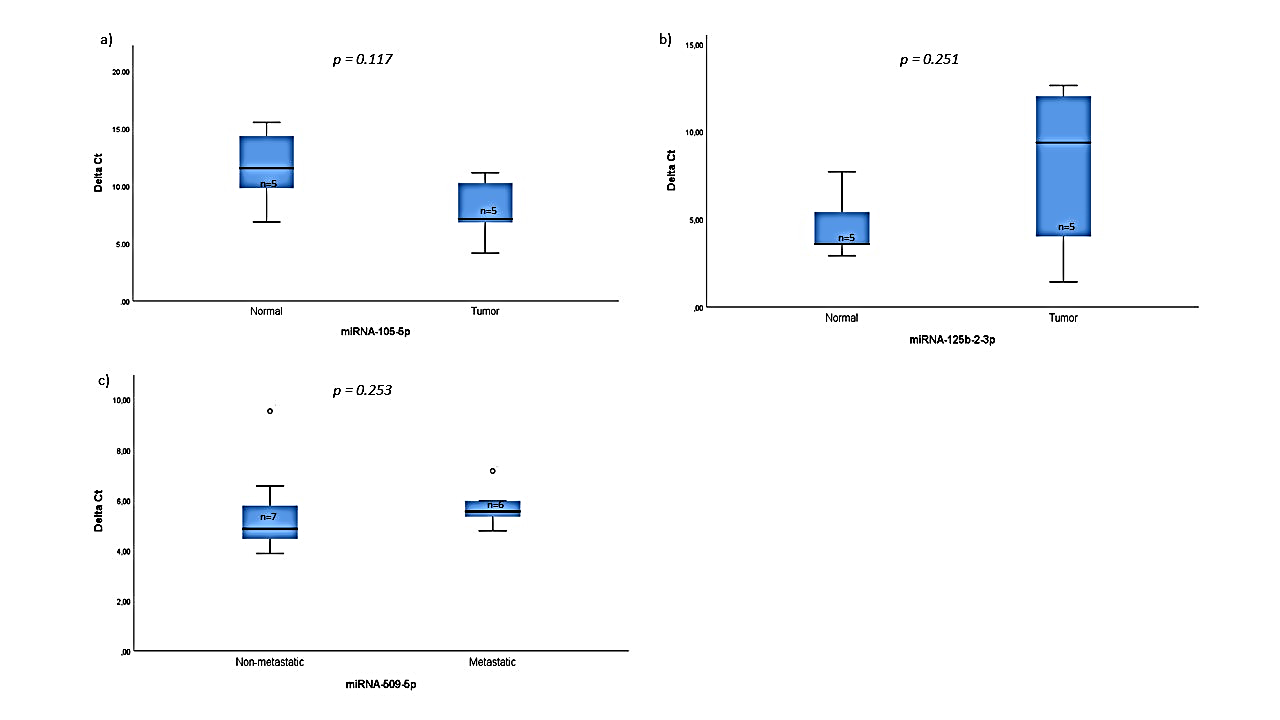 |
| --- |
| Figure S4. miRNAs without significant differences as determined by TaqMan® RT-qPCR (p ≤ 0.05). (a) and (b)Mean ΔCt value of (HPV-negative) usual PSCC and normal tissue samples; (c) mean ΔCt value of metastatic versus non-metastatic usual (HPV-negative) tumors; Lower ΔCt implays higher expression level. Mann-Whitney U test was used to determine p-values |
