# Supplementary material for: miRNA Expression Characterizes Histological Subtypes and Metastasis in Penile Squamous Cell Carcinoma
Source: Cancers (Basel). 2021 Mar 23;13(6):1480. doi: 10.3390/cancers13061480 (PMC8004785; doi:10.3390/cancers13061480)
Supplement: Supplementary file 1 [file cancers-13-01480-s001.zip › cancers-1116103-supp/Table S2.docx]

Table S2. Significantly differentially expressed miRNAs in HPV-positive versus HPV-negative PSCC.

| Systematic name | p-value | q-value | Fold change |
| --- | --- | --- | --- |
| hsa-miR-5696 | 0.005 | 0.018 | 3.214 |
| hsa-miR-3925-5p | 0.003 | 0.018 | 2.851 |
| hsa-miR-1273c | 0.001 | 0.018 | 2.725 |
| hsa-miR-4522 | 0.005 | 0.018 | 2.671 |
| hsa-miR-6847-5p | 0.004 | 0.018 | 2.532 |
| hsa-miR-6857-5p | 0.006 | 0.020 | 2.236 |
| hsa-miR-370-3p | 0.009 | 0.026 | 2.115 |
| hsa-miR-4758-5p | 0.005 | 0.019 | 2.069 |
| hsa-miR-5100 | 0.004 | 0.018 | 0.500 |
| hsa-miR-20b-3p | 0.008 | 0.024 | 0.499 |
| hsa-miR-8079 | 0.005 | 0.019 | 0.499 |
| hsa-miR-138-1-3p | 0.008 | 0.023 | 0.494 |
| hsa-miR-6884-5p | 0.009 | 0.026 | 0.494 |
| hsa-miR-6501-5p | 0.009 | 0.025 | 0.494 |
| hsa-miR-8070 | 0.010 | 0.027 | 0.493 |
| hsa-miR-6802-3p | 0.007 | 0.022 | 0.493 |
| hsa-miR-608 | 0.009 | 0.026 | 0.492 |
| hsa-miR-8054 | 0.007 | 0.022 | 0.492 |
| hsa-miR-3613-3p | 0.005 | 0.019 | 0.492 |
| hsa-miR-6508-5p | 0.002 | 0.018 | 0.491 |
| hsa-miR-873-5p | 0.010 | 0.027 | 0.491 |
| hsa-miR-1298-3p | 0.007 | 0.022 | 0.491 |
| hsa-miR-885-3p | 0.008 | 0.023 | 0.491 |
| hsa-miR-129-2-3p | 0.008 | 0.023 | 0.491 |
| hsa-miR-3148 | 0.009 | 0.026 | 0.490 |
| hsa-miR-1234-3p | 0.002 | 0.018 | 0.490 |
| hsa-miR-8080 | 0.008 | 0.024 | 0.490 |
| hsa-miR-148a-5p | 0.009 | 0.026 | 0.490 |
| hsa-miR-520f-3p | 0.009 | 0.026 | 0.489 |
| hsa-miR-30d-3p | 0.004 | 0.018 | 0.488 |
| hsa-miR-433-3p | 0.009 | 0.026 | 0.487 |
| hsa-miR-3064-5p | 0.008 | 0.024 | 0.487 |
| hsa-miR-6821-3p | 0.008 | 0.023 | 0.486 |
| hsa-miR-3529-3p | 0.009 | 0.026 | 0.485 |
| hsa-miR-4747-5p | 0.008 | 0.023 | 0.484 |
| hsa-miR-889-5p | 0.008 | 0.024 | 0.483 |
| hsa-miR-5692a | 0.008 | 0.023 | 0.483 |
| hsa-miR-7160-5p | 0.006 | 0.020 | 0.483 |
| hsa-miR-548b-5p | 0.009 | 0.025 | 0.482 |
| hsa-miR-300 | 0.004 | 0.018 | 0.481 |
| hsa-miR-548ae-3p | 0.009 | 0.026 | 0.481 |
| hsa-miR-4764-5p | 0.007 | 0.021 | 0.480 |
| hsa-miR-548ad-5p | 0.008 | 0.024 | 0.479 |
| hsa-miR-6504-3p | 0.009 | 0.025 | 0.478 |
| hsa-miR-4754 | 0.008 | 0.023 | 0.477 |
| hsa-miR-3944-3p | 0.007 | 0.021 | 0.476 |
| hsa-miR-331-5p | 0.003 | 0.018 | 0.475 |
| hsa-miR-5698 | 0.007 | 0.022 | 0.475 |
| hsa-miR-219a-2-3p | 0.007 | 0.022 | 0.474 |
| hsa-miR-8083 | 0.005 | 0.018 | 0.474 |
| hsa-miR-645 | 0.004 | 0.018 | 0.474 |
| hsa-miR-6871-3p | 0.004 | 0.018 | 0.474 |
| hsa-miR-517-5p | 0.007 | 0.023 | 0.473 |
| hsa-miR-675-5p | 0.008 | 0.023 | 0.473 |
| hsa-miR-4766-5p | 0.007 | 0.022 | 0.472 |
| hsa-miR-4440 | 0.006 | 0.020 | 0.472 |
| hsa-miR-2116-5p | 0.006 | 0.020 | 0.472 |
| hsa-miR-518f-5p | 0.008 | 0.024 | 0.472 |
| hsa-miR-3186-5p | 0.008 | 0.024 | 0.471 |
| hsa-miR-548bb-3p | 0.010 | 0.027 | 0.471 |
| hsa-miR-4266 | 0.007 | 0.022 | 0.470 |
| hsa-miR-4653-5p | 0.004 | 0.018 | 0.470 |
| hsa-miR-4293 | 0.006 | 0.020 | 0.470 |
| hsa-miR-548e-3p | 0.006 | 0.019 | 0.470 |
| hsa-miR-7703 | 0.004 | 0.018 | 0.469 |
| hsa-miR-192-3p | 0.007 | 0.021 | 0.469 |
| hsa-miR-6746-3p | 0.006 | 0.020 | 0.468 |
| hsa-miR-135b-3p | 0.005 | 0.019 | 0.468 |
| hsa-miR-4276 | 0.008 | 0.023 | 0.467 |
| hsa-miR-6895-3p | 0.009 | 0.026 | 0.467 |
| hsa-miR-6854-3p | 0.006 | 0.020 | 0.467 |
| hsa-miR-548aw | 0.006 | 0.020 | 0.467 |
| hsa-miR-3191-3p | 0.006 | 0.019 | 0.467 |
| hsa-miR-589-3p | 0.008 | 0.023 | 0.467 |
| hsa-miR-5000-3p | 0.006 | 0.020 | 0.467 |
| hsa-miR-92a-1-5p | 0.007 | 0.022 | 0.466 |
| hsa-let-7a-3p | 0.006 | 0.019 | 0.466 |
| hsa-miR-379-3p | 0.009 | 0.026 | 0.466 |
| hsa-miR-2682-3p | 0.007 | 0.022 | 0.466 |
| hsa-miR-562 | 0.009 | 0.026 | 0.465 |
| hsa-miR-148b-5p | 0.006 | 0.020 | 0.465 |
| hsa-miR-6773-5p | 0.006 | 0.021 | 0.465 |
| hsa-miR-584-3p | 0.004 | 0.018 | 0.465 |
| hsa-miR-3074-5p | 0.007 | 0.021 | 0.464 |
| hsa-miR-500b-3p | 0.007 | 0.022 | 0.464 |
| hsa-miR-135a-5p | 0.007 | 0.023 | 0.464 |
| hsa-miR-4677-5p | 0.006 | 0.019 | 0.463 |
| hsa-miR-6734-3p | 0.008 | 0.023 | 0.463 |
| hsa-miR-4520-5p | 0.008 | 0.023 | 0.463 |
| hsa-miR-5000-5p | 0.002 | 0.018 | 0.463 |
| hsa-miR-649 | 0.007 | 0.022 | 0.463 |
| hsa-miR-4652-3p | 0.004 | 0.018 | 0.462 |
| hsa-miR-6818-5p | 0.004 | 0.018 | 0.462 |
| hsa-miR-5089-3p | 0.006 | 0.019 | 0.462 |
| hsa-miR-4520-3p | 0.008 | 0.023 | 0.462 |
| hsa-miR-4791 | 0.008 | 0.023 | 0.462 |
| hsa-miR-5680 | 0.008 | 0.024 | 0.462 |
| hsa-miR-7853-5p | 0.005 | 0.019 | 0.461 |
| hsa-miR-323a-5p | 0.005 | 0.018 | 0.461 |
| hsa-miR-7112-3p | 0.008 | 0.023 | 0.461 |
| hsa-miR-5691 | 0.005 | 0.019 | 0.461 |
| hsa-miR-3144-3p | 0.008 | 0.023 | 0.460 |
| hsa-miR-193a-5p | 0.010 | 0.027 | 0.460 |
| hsa-miR-551a | 0.007 | 0.022 | 0.460 |
| hsa-miR-4797-3p | 0.004 | 0.018 | 0.459 |
| hsa-miR-548az-5p | 0.010 | 0.027 | 0.459 |
| hsa-miR-541-3p | 0.006 | 0.020 | 0.458 |
| hsa-miR-548c-3p | 0.007 | 0.022 | 0.458 |
| hsa-miR-6834-5p | 0.004 | 0.018 | 0.458 |
| hsa-miR-4423-3p | 0.003 | 0.018 | 0.458 |
| hsa-miR-1287-3p | 0.003 | 0.018 | 0.458 |
| hsa-miR-371b-3p | 0.003 | 0.018 | 0.458 |
| hsa-miR-875-3p | 0.005 | 0.018 | 0.457 |
| hsa-miR-152-5p | 0.009 | 0.025 | 0.457 |
| hsa-miR-3200-3p | 0.005 | 0.018 | 0.457 |
| hsa-miR-4714-3p | 0.004 | 0.018 | 0.457 |
| hsa-miR-3689b-3p | 0.006 | 0.020 | 0.457 |
| hsa-miR-29a-5p | 0.007 | 0.021 | 0.456 |
| hsa-miR-7843-5p | 0.004 | 0.018 | 0.456 |
| hsa-miR-6788-3p | 0.006 | 0.020 | 0.456 |
| hsa-miR-4774-3p | 0.005 | 0.018 | 0.456 |
| hsa-miR-1251-3p | 0.007 | 0.022 | 0.456 |
| hsa-miR-5588-5p | 0.008 | 0.024 | 0.456 |
| hsa-miR-27b-5p | 0.006 | 0.019 | 0.455 |
| hsa-miR-676-5p | 0.005 | 0.018 | 0.455 |
| hsa-miR-6721-5p | 0.005 | 0.019 | 0.455 |
| hsa-miR-4309 | 0.005 | 0.019 | 0.455 |
| hsa-miR-6505-5p | 0.008 | 0.024 | 0.455 |
| hsa-miR-545-3p | 0.004 | 0.018 | 0.455 |
| hsa-miR-661 | 0.004 | 0.018 | 0.454 |
| hsa-miR-122-5p | 0.007 | 0.022 | 0.454 |
| hsa-miR-302e | 0.007 | 0.022 | 0.454 |
| hsa-miR-4273 | 0.003 | 0.018 | 0.454 |
| hsa-miR-147b | 0.007 | 0.022 | 0.453 |
| hsa-miR-3194-3p | 0.006 | 0.019 | 0.453 |
| hsa-miR-6506-5p | 0.008 | 0.024 | 0.452 |
| hsa-miR-506-5p | 0.003 | 0.018 | 0.452 |
| hsa-miR-4796-3p | 0.004 | 0.018 | 0.452 |
| hsa-miR-4426 | 0.006 | 0.020 | 0.452 |
| hsa-miR-3065-3p | 0.005 | 0.018 | 0.452 |
| hsa-miR-6764-3p | 0.005 | 0.018 | 0.452 |
| hsa-miR-615-5p | 0.005 | 0.018 | 0.452 |
| hsa-miR-222-5p | 0.004 | 0.018 | 0.452 |
| hsa-miR-548j-5p | 0.007 | 0.022 | 0.452 |
| hsa-miR-612 | 0.006 | 0.020 | 0.452 |
| hsa-miR-7158-5p | 0.004 | 0.018 | 0.451 |
| hsa-miR-6808-3p | 0.004 | 0.018 | 0.451 |
| hsa-miR-7154-3p | 0.003 | 0.018 | 0.451 |
| hsa-miR-2355-3p | 0.001 | 0.018 | 0.451 |
| hsa-miR-548ap-3p | 0.006 | 0.021 | 0.451 |
| hsa-miR-5088-3p | 0.003 | 0.018 | 0.451 |
| hsa-miR-3183 | 0.005 | 0.018 | 0.450 |
| hsa-miR-4445-3p | 0.006 | 0.019 | 0.450 |
| hsa-miR-548ar-5p | 0.006 | 0.020 | 0.450 |
| hsa-miR-6838-3p | 0.007 | 0.021 | 0.450 |
| hsa-miR-4708-3p | 0.006 | 0.020 | 0.449 |
| hsa-miR-4801 | 0.003 | 0.018 | 0.449 |
| hsa-miR-548ao-3p | 0.006 | 0.020 | 0.449 |
| hsa-miR-635 | 0.004 | 0.018 | 0.449 |
| hsa-miR-3939 | 0.003 | 0.018 | 0.449 |
| hsa-miR-6731-5p | 0.004 | 0.018 | 0.448 |
| hsa-miR-647 | 0.008 | 0.024 | 0.448 |
| hsa-miR-6832-5p | 0.005 | 0.019 | 0.448 |
| hsa-miR-548x-3p | 0.004 | 0.018 | 0.448 |
| hsa-miR-578 | 0.006 | 0.019 | 0.448 |
| hsa-miR-380-5p | 0.004 | 0.018 | 0.448 |
| hsa-miR-6853-3p | 0.005 | 0.019 | 0.448 |
| hsa-miR-3192-5p | 0.005 | 0.019 | 0.448 |
| hsa-miR-218-2-3p | 0.003 | 0.018 | 0.448 |
| hsa-miR-6715b-5p | 0.004 | 0.018 | 0.447 |
| hsa-miR-3116 | 0.006 | 0.020 | 0.447 |
| hsa-miR-6817-3p | 0.003 | 0.018 | 0.447 |
| hsa-miR-4278 | 0.004 | 0.018 | 0.447 |
| hsa-miR-5707 | 0.004 | 0.018 | 0.447 |
| hsa-miR-5587-3p | 0.006 | 0.020 | 0.446 |
| hsa-miR-146b-3p | 0.006 | 0.019 | 0.446 |
| hsa-miR-410-5p | 0.005 | 0.018 | 0.446 |
| hsa-miR-2115-5p | 0.004 | 0.018 | 0.446 |
| hsa-miR-412-3p | 0.005 | 0.019 | 0.446 |
| hsa-miR-514b-3p | 0.004 | 0.018 | 0.446 |
| hsa-miR-3144-5p | 0.005 | 0.019 | 0.446 |
| hsa-miR-548ap-5p | 0.006 | 0.019 | 0.446 |
| hsa-miR-7153-3p | 0.005 | 0.019 | 0.446 |
| hsa-miR-4277 | 0.003 | 0.018 | 0.446 |
| hsa-miR-3157-5p | 0.005 | 0.018 | 0.446 |
| hsa-miR-33b-5p | 0.007 | 0.021 | 0.445 |
| hsa-miR-548j-3p | 0.006 | 0.020 | 0.445 |
| hsa-miR-1206 | 0.007 | 0.022 | 0.445 |
| hsa-miR-1282 | 0.007 | 0.021 | 0.445 |
| hsa-miR-1468-3p | 0.004 | 0.018 | 0.445 |
| hsa-miR-3913-3p | 0.005 | 0.019 | 0.445 |
| hsa-miR-577 | 0.005 | 0.019 | 0.444 |
| hsa-miR-4782-3p | 0.006 | 0.020 | 0.444 |
| hsa-miR-488-3p | 0.010 | 0.027 | 0.444 |
| hsa-miR-5001-3p | 0.005 | 0.019 | 0.444 |
| hsa-miR-1197 | 0.003 | 0.018 | 0.444 |
| hsa-miR-6764-5p | 0.005 | 0.018 | 0.444 |
| hsa-miR-5004-3p | 0.004 | 0.018 | 0.444 |
| hsa-miR-604 | 0.003 | 0.018 | 0.444 |
| hsa-miR-3655 | 0.006 | 0.020 | 0.444 |
| hsa-miR-4683 | 0.003 | 0.018 | 0.444 |
| hsa-miR-1263 | 0.004 | 0.018 | 0.443 |
| hsa-miR-4482-5p | 0.004 | 0.018 | 0.443 |
| hsa-miR-593-5p | 0.004 | 0.018 | 0.443 |
| hsa-miR-571 | 0.006 | 0.021 | 0.443 |
| hsa-miR-4536-5p | 0.007 | 0.022 | 0.443 |
| hsa-miR-4677-3p | 0.005 | 0.019 | 0.442 |
| hsa-miR-3928-3p | 0.006 | 0.019 | 0.442 |
| hsa-miR-191-5p | 0.003 | 0.018 | 0.442 |
| hsa-miR-8058 | 0.005 | 0.019 | 0.442 |
| hsa-miR-4256 | 0.004 | 0.018 | 0.442 |
| hsa-miR-5004-5p | 0.004 | 0.018 | 0.442 |
| hsa-miR-1180-5p | 0.002 | 0.018 | 0.442 |
| hsa-miR-4301 | 0.005 | 0.018 | 0.442 |
| hsa-miR-6512-3p | 0.005 | 0.018 | 0.442 |
| hsa-miR-4691-3p | 0.005 | 0.018 | 0.441 |
| hsa-miR-548aj-3p | 0.006 | 0.020 | 0.441 |
| hsa-miR-3136-3p | 0.006 | 0.019 | 0.441 |
| hsa-miR-4742-3p | 0.004 | 0.018 | 0.441 |
| hsa-miR-4762-3p | 0.005 | 0.019 | 0.441 |
| hsa-miR-6853-5p | 0.003 | 0.018 | 0.441 |
| hsa-miR-586 | 0.006 | 0.021 | 0.441 |
| hsa-miR-4525 | 0.004 | 0.018 | 0.441 |
| hsa-miR-576-3p | 0.004 | 0.018 | 0.441 |
| hsa-miR-548i | 0.008 | 0.023 | 0.440 |
| hsa-miR-4650-3p | 0.004 | 0.018 | 0.440 |
| hsa-miR-3606-5p | 0.007 | 0.022 | 0.440 |
| hsa-miR-6811-3p | 0.005 | 0.018 | 0.440 |
| hsa-miR-3689d | 0.004 | 0.018 | 0.440 |
| hsa-miR-3136-5p | 0.004 | 0.018 | 0.440 |
| hsa-miR-3145-5p | 0.009 | 0.025 | 0.439 |
| hsa-miR-4302 | 0.004 | 0.018 | 0.439 |
| hsa-miR-554 | 0.004 | 0.018 | 0.439 |
| hsa-miR-1292-5p | 0.002 | 0.018 | 0.439 |
| hsa-miR-4474-3p | 0.004 | 0.018 | 0.439 |
| hsa-miR-524-3p | 0.004 | 0.018 | 0.439 |
| hsa-miR-8061 | 0.005 | 0.018 | 0.438 |
| hsa-miR-653-5p | 0.006 | 0.020 | 0.438 |
| hsa-miR-5591-5p | 0.005 | 0.018 | 0.438 |
| hsa-miR-5688 | 0.005 | 0.018 | 0.438 |
| hsa-miR-545-5p | 0.005 | 0.018 | 0.438 |
| hsa-miR-548aj-5p | 0.008 | 0.023 | 0.438 |
| hsa-miR-3180 | 0.004 | 0.018 | 0.438 |
| hsa-miR-122-3p | 0.003 | 0.018 | 0.438 |
| hsa-miR-218-1-3p | 0.004 | 0.018 | 0.438 |
| hsa-miR-6829-3p | 0.003 | 0.018 | 0.437 |
| hsa-miR-1322 | 0.005 | 0.019 | 0.437 |
| hsa-miR-4717-5p | 0.004 | 0.018 | 0.437 |
| hsa-miR-3184-5p | 0.004 | 0.018 | 0.437 |
| hsa-miR-8053 | 0.003 | 0.018 | 0.437 |
| hsa-miR-6783-5p | 0.005 | 0.018 | 0.437 |
| hsa-miR-6078 | 0.003 | 0.018 | 0.437 |
| hsa-miR-4795-5p | 0.005 | 0.018 | 0.436 |
| hsa-miR-188-3p | 0.004 | 0.018 | 0.436 |
| hsa-miR-1297 | 0.004 | 0.018 | 0.436 |
| hsa-miR-3120-5p | 0.005 | 0.018 | 0.436 |
| hsa-miR-6755-5p | 0.004 | 0.018 | 0.436 |
| hsa-miR-106a-3p | 0.006 | 0.020 | 0.436 |
| hsa-miR-4779 | 0.003 | 0.018 | 0.436 |
| hsa-miR-202-5p | 0.008 | 0.023 | 0.436 |
| hsa-miR-1304-5p | 0.005 | 0.018 | 0.436 |
| hsa-miR-6504-5p | 0.005 | 0.018 | 0.436 |
| hsa-miR-892c-3p | 0.003 | 0.018 | 0.436 |
| hsa-miR-4661-3p | 0.005 | 0.019 | 0.436 |
| hsa-miR-892a | 0.005 | 0.019 | 0.435 |
| hsa-miR-4693-5p | 0.006 | 0.019 | 0.435 |
| hsa-miR-520a-5p | 0.004 | 0.018 | 0.435 |
| hsa-miR-5694 | 0.003 | 0.018 | 0.435 |
| hsa-miR-3126-5p | 0.003 | 0.018 | 0.435 |
| hsa-miR-3166 | 0.003 | 0.018 | 0.435 |
| hsa-miR-5092 | 0.006 | 0.019 | 0.435 |
| hsa-miR-520h | 0.005 | 0.018 | 0.435 |
| hsa-miR-6888-3p | 0.004 | 0.018 | 0.435 |
| hsa-miR-4437 | 0.004 | 0.018 | 0.435 |
| hsa-miR-181b-3p | 0.005 | 0.019 | 0.434 |
| hsa-miR-548a-5p | 0.005 | 0.019 | 0.434 |
| hsa-miR-3910 | 0.005 | 0.019 | 0.434 |
| hsa-miR-4744 | 0.004 | 0.018 | 0.434 |
| hsa-miR-761 | 0.004 | 0.018 | 0.434 |
| hsa-miR-3689a-3p | 0.004 | 0.018 | 0.434 |
| hsa-miR-4756-5p | 0.004 | 0.018 | 0.434 |
| hsa-miR-892c-5p | 0.003 | 0.018 | 0.434 |
| hsa-miR-6514-5p | 0.004 | 0.018 | 0.434 |
| hsa-miR-3664-3p | 0.004 | 0.018 | 0.434 |
| hsa-miR-548at-5p | 0.005 | 0.019 | 0.434 |
| hsa-miR-514a-5p | 0.003 | 0.018 | 0.434 |
| hsa-miR-942-3p | 0.004 | 0.018 | 0.434 |
| hsa-miR-548au-5p | 0.006 | 0.020 | 0.434 |
| hsa-miR-4764-3p | 0.003 | 0.018 | 0.434 |
| hsa-miR-4521 | 0.004 | 0.018 | 0.434 |
| hsa-miR-4477b | 0.004 | 0.018 | 0.433 |
| hsa-miR-6811-5p | 0.004 | 0.018 | 0.433 |
| hsa-miR-4528 | 0.005 | 0.018 | 0.433 |
| hsa-miR-4724-3p | 0.005 | 0.018 | 0.433 |
| hsa-miR-548av-3p | 0.003 | 0.018 | 0.433 |
| hsa-miR-641 | 0.003 | 0.018 | 0.433 |
| hsa-miR-6876-3p | 0.005 | 0.018 | 0.433 |
| hsa-miR-301b-5p | 0.003 | 0.018 | 0.433 |
| hsa-miR-6781-3p | 0.005 | 0.018 | 0.433 |
| hsa-miR-4420 | 0.005 | 0.019 | 0.433 |
| hsa-miR-7705 | 0.004 | 0.018 | 0.433 |
| hsa-miR-7973 | 0.005 | 0.018 | 0.433 |
| hsa-miR-302d-5p | 0.004 | 0.018 | 0.432 |
| hsa-miR-4504 | 0.004 | 0.018 | 0.432 |
| hsa-miR-4524b-3p | 0.003 | 0.018 | 0.432 |
| hsa-miR-370-5p | 0.004 | 0.018 | 0.432 |
| hsa-miR-4772-3p | 0.004 | 0.018 | 0.432 |
| hsa-miR-6767-3p | 0.003 | 0.018 | 0.432 |
| hsa-miR-6715a-3p | 0.004 | 0.018 | 0.432 |
| hsa-miR-4699-5p | 0.004 | 0.018 | 0.432 |
| hsa-miR-4523 | 0.004 | 0.018 | 0.432 |
| hsa-miR-605-3p | 0.004 | 0.018 | 0.432 |
| hsa-miR-4733-3p | 0.004 | 0.018 | 0.431 |
| hsa-miR-216b-5p | 0.004 | 0.018 | 0.431 |
| hsa-miR-449b-5p | 0.005 | 0.018 | 0.431 |
| hsa-miR-4762-5p | 0.005 | 0.018 | 0.431 |
| hsa-miR-6768-3p | 0.002 | 0.018 | 0.431 |
| hsa-miR-4671-3p | 0.004 | 0.018 | 0.431 |
| hsa-miR-450a-1-3p | 0.004 | 0.018 | 0.431 |
| hsa-miR-3127-3p | 0.004 | 0.018 | 0.431 |
| hsa-miR-382-3p | 0.006 | 0.019 | 0.431 |
| hsa-miR-5692b | 0.005 | 0.018 | 0.431 |
| hsa-miR-8084 | 0.004 | 0.018 | 0.431 |
| hsa-miR-6878-5p | 0.004 | 0.018 | 0.430 |
| hsa-miR-5702 | 0.004 | 0.018 | 0.430 |
| hsa-miR-548av-5p | 0.004 | 0.018 | 0.430 |
| hsa-miR-329-5p | 0.003 | 0.018 | 0.430 |
| hsa-miR-146a-3p | 0.008 | 0.023 | 0.430 |
| hsa-miR-548f-3p | 0.004 | 0.018 | 0.430 |
| hsa-miR-217 | 0.004 | 0.018 | 0.430 |
| hsa-miR-488-5p | 0.004 | 0.018 | 0.430 |
| hsa-miR-4772-5p | 0.005 | 0.018 | 0.430 |
| hsa-miR-4799-5p | 0.004 | 0.018 | 0.430 |
| hsa-miR-499b-3p | 0.003 | 0.018 | 0.430 |
| hsa-miR-96-3p | 0.005 | 0.018 | 0.430 |
| hsa-miR-3919 | 0.004 | 0.018 | 0.430 |
| hsa-miR-3126-3p | 0.003 | 0.018 | 0.430 |
| hsa-miR-616-5p | 0.005 | 0.018 | 0.430 |
| hsa-miR-92a-2-5p | 0.004 | 0.018 | 0.430 |
| hsa-miR-5189-3p | 0.006 | 0.020 | 0.430 |
| hsa-miR-2681-3p | 0.005 | 0.018 | 0.430 |
| hsa-miR-548an | 0.006 | 0.019 | 0.430 |
| hsa-miR-2467-5p | 0.004 | 0.018 | 0.429 |
| hsa-miR-4686 | 0.003 | 0.018 | 0.429 |
| hsa-miR-6501-3p | 0.003 | 0.018 | 0.429 |
| hsa-miR-363-5p | 0.005 | 0.018 | 0.429 |
| hsa-miR-8068 | 0.005 | 0.018 | 0.429 |
| hsa-miR-3941 | 0.004 | 0.018 | 0.429 |
| hsa-miR-4693-3p | 0.006 | 0.019 | 0.429 |
| hsa-miR-523-3p | 0.003 | 0.018 | 0.429 |
| hsa-miR-4777-5p | 0.004 | 0.018 | 0.429 |
| hsa-miR-4727-5p | 0.005 | 0.018 | 0.429 |
| hsa-miR-668-5p | 0.002 | 0.018 | 0.429 |
| hsa-miR-3668 | 0.004 | 0.018 | 0.428 |
| hsa-miR-450a-2-3p | 0.004 | 0.018 | 0.428 |
| hsa-miR-383-3p | 0.003 | 0.018 | 0.428 |
| hsa-miR-6852-5p | 0.003 | 0.018 | 0.428 |
| hsa-miR-590-3p | 0.004 | 0.018 | 0.428 |
| hsa-miR-600 | 0.004 | 0.018 | 0.428 |
| hsa-miR-216a-5p | 0.002 | 0.018 | 0.428 |
| hsa-miR-4467 | 0.004 | 0.018 | 0.428 |
| hsa-miR-153-3p | 0.008 | 0.023 | 0.428 |
| hsa-miR-1912 | 0.004 | 0.018 | 0.427 |
| hsa-miR-4469 | 0.003 | 0.018 | 0.427 |
| hsa-miR-187-3p | 0.005 | 0.018 | 0.427 |
| hsa-miR-450b-3p | 0.006 | 0.020 | 0.427 |
| hsa-miR-4703-5p | 0.005 | 0.018 | 0.427 |
| hsa-miR-452-3p | 0.004 | 0.018 | 0.427 |
| hsa-miR-1255a | 0.004 | 0.018 | 0.427 |
| hsa-miR-6077 | 0.003 | 0.018 | 0.427 |
| hsa-miR-3936 | 0.004 | 0.018 | 0.427 |
| hsa-miR-8062 | 0.004 | 0.018 | 0.427 |
| hsa-miR-4729 | 0.003 | 0.018 | 0.427 |
| hsa-miR-890 | 0.002 | 0.018 | 0.427 |
| hsa-miR-708-3p | 0.003 | 0.018 | 0.427 |
| hsa-miR-1257 | 0.003 | 0.018 | 0.427 |
| hsa-miR-4778-3p | 0.004 | 0.018 | 0.427 |
| hsa-miR-4663 | 0.004 | 0.018 | 0.427 |
| hsa-miR-589-5p | 0.004 | 0.018 | 0.426 |
| hsa-miR-219a-1-3p | 0.004 | 0.018 | 0.426 |
| hsa-miR-3165 | 0.004 | 0.018 | 0.426 |
| hsa-miR-24-2-5p | 0.004 | 0.018 | 0.426 |
| hsa-miR-548al | 0.004 | 0.018 | 0.426 |
| hsa-miR-1231 | 0.004 | 0.018 | 0.426 |
| hsa-miR-548ar-3p | 0.004 | 0.018 | 0.426 |
| hsa-miR-548f-5p | 0.004 | 0.018 | 0.426 |
| hsa-miR-4684-5p | 0.003 | 0.018 | 0.426 |
| hsa-miR-1272 | 0.004 | 0.018 | 0.426 |
| hsa-miR-548n | 0.005 | 0.019 | 0.426 |
| hsa-miR-4637 | 0.003 | 0.018 | 0.426 |
| hsa-miR-1193 | 0.003 | 0.018 | 0.426 |
| hsa-miR-3168 | 0.004 | 0.018 | 0.426 |
| hsa-miR-581 | 0.005 | 0.019 | 0.426 |
| hsa-miR-3974 | 0.006 | 0.019 | 0.426 |
| hsa-miR-6128 | 0.004 | 0.018 | 0.426 |
| hsa-miR-323a-3p | 0.004 | 0.018 | 0.426 |
| hsa-miR-5586-3p | 0.004 | 0.018 | 0.425 |
| hsa-miR-4424 | 0.005 | 0.018 | 0.425 |
| hsa-miR-4752 | 0.004 | 0.018 | 0.425 |
| hsa-miR-4781-3p | 0.004 | 0.018 | 0.425 |
| hsa-miR-1178-5p | 0.004 | 0.018 | 0.425 |
| hsa-miR-5186 | 0.004 | 0.018 | 0.425 |
| hsa-miR-4745-3p | 0.003 | 0.018 | 0.425 |
| hsa-miR-6841-5p | 0.005 | 0.018 | 0.425 |
| hsa-miR-4735-5p | 0.004 | 0.018 | 0.425 |
| hsa-miR-302a-5p | 0.004 | 0.018 | 0.425 |
| hsa-miR-5695 | 0.004 | 0.018 | 0.425 |
| hsa-miR-4434 | 0.004 | 0.018 | 0.425 |
| hsa-miR-6835-5p | 0.003 | 0.018 | 0.425 |
| hsa-miR-3677-5p | 0.004 | 0.018 | 0.425 |
| hsa-miR-3142 | 0.004 | 0.018 | 0.425 |
| hsa-miR-6744-3p | 0.005 | 0.018 | 0.425 |
| hsa-miR-1293 | 0.002 | 0.018 | 0.424 |
| hsa-miR-4715-3p | 0.004 | 0.018 | 0.424 |
| hsa-miR-6513-5p | 0.003 | 0.018 | 0.424 |
| hsa-miR-4285 | 0.004 | 0.018 | 0.424 |
| hsa-miR-4464 | 0.004 | 0.018 | 0.424 |
| hsa-miR-1295b-5p | 0.005 | 0.018 | 0.424 |
| hsa-miR-497-3p | 0.004 | 0.018 | 0.424 |
| hsa-miR-5687 | 0.004 | 0.018 | 0.424 |
| hsa-miR-4264 | 0.003 | 0.018 | 0.424 |
| hsa-miR-8081 | 0.004 | 0.018 | 0.424 |
| hsa-miR-1205 | 0.003 | 0.018 | 0.424 |
| hsa-miR-555 | 0.002 | 0.018 | 0.424 |
| hsa-miR-3129-5p | 0.003 | 0.018 | 0.424 |
| hsa-miR-4711-3p | 0.004 | 0.018 | 0.424 |
| hsa-miR-5582-5p | 0.004 | 0.018 | 0.424 |
| hsa-miR-6842-3p | 0.003 | 0.018 | 0.424 |
| hsa-miR-5091 | 0.003 | 0.018 | 0.423 |
| hsa-miR-3611 | 0.004 | 0.018 | 0.423 |
| hsa-miR-3681-5p | 0.003 | 0.018 | 0.423 |
| hsa-miR-8076 | 0.004 | 0.018 | 0.423 |
| hsa-miR-29b-2-5p | 0.004 | 0.018 | 0.423 |
| hsa-miR-4491 | 0.004 | 0.018 | 0.423 |
| hsa-miR-1279 | 0.005 | 0.018 | 0.423 |
| hsa-miR-6742-5p | 0.003 | 0.018 | 0.423 |
| hsa-miR-6813-5p | 0.002 | 0.018 | 0.423 |
| hsa-miR-6843-3p | 0.003 | 0.018 | 0.423 |
| hsa-miR-92b-5p | 0.003 | 0.018 | 0.423 |
| hsa-miR-4742-5p | 0.003 | 0.018 | 0.423 |
| hsa-miR-3671 | 0.003 | 0.018 | 0.423 |
| hsa-miR-4781-5p | 0.003 | 0.018 | 0.423 |
| hsa-miR-433-5p | 0.003 | 0.018 | 0.422 |
| hsa-miR-6079 | 0.003 | 0.018 | 0.422 |
| hsa-miR-5590-3p | 0.004 | 0.018 | 0.422 |
| hsa-miR-182-3p | 0.003 | 0.018 | 0.422 |
| hsa-miR-133a-5p | 0.005 | 0.018 | 0.422 |
| hsa-miR-1908-5p | 0.003 | 0.018 | 0.422 |
| hsa-miR-6770-5p | 0.004 | 0.018 | 0.422 |
| hsa-miR-3927-3p | 0.004 | 0.018 | 0.422 |
| hsa-miR-651-5p | 0.004 | 0.018 | 0.422 |
| hsa-miR-4292 | 0.003 | 0.018 | 0.422 |
| hsa-miR-1245a | 0.007 | 0.022 | 0.422 |
| hsa-miR-3925-3p | 0.009 | 0.026 | 0.422 |
| hsa-miR-499a-3p | 0.004 | 0.018 | 0.422 |
| hsa-miR-548w | 0.005 | 0.019 | 0.422 |
| hsa-miR-153-5p | 0.007 | 0.021 | 0.422 |
| hsa-miR-181b-2-3p | 0.002 | 0.018 | 0.422 |
| hsa-miR-599 | 0.003 | 0.018 | 0.422 |
| hsa-miR-208a-3p | 0.003 | 0.018 | 0.421 |
| hsa-miR-3942-5p | 0.005 | 0.018 | 0.421 |
| hsa-miR-556-5p | 0.004 | 0.018 | 0.421 |
| hsa-miR-466 | 0.002 | 0.018 | 0.421 |
| hsa-miR-4724-5p | 0.003 | 0.018 | 0.421 |
| hsa-miR-3973 | 0.004 | 0.018 | 0.421 |
| hsa-miR-3688-5p | 0.002 | 0.018 | 0.421 |
| hsa-miR-646 | 0.002 | 0.018 | 0.421 |
| hsa-miR-7978 | 0.003 | 0.018 | 0.421 |
| hsa-miR-4751 | 0.003 | 0.018 | 0.421 |
| hsa-miR-5706 | 0.003 | 0.018 | 0.421 |
| hsa-miR-4649-5p | 0.003 | 0.018 | 0.421 |
| hsa-miR-100-3p | 0.004 | 0.018 | 0.421 |
| hsa-miR-922 | 0.003 | 0.018 | 0.420 |
| hsa-miR-302b-5p | 0.004 | 0.018 | 0.420 |
| hsa-miR-3691-5p | 0.003 | 0.018 | 0.420 |
| hsa-miR-4774-5p | 0.003 | 0.018 | 0.420 |
| hsa-miR-3909 | 0.003 | 0.018 | 0.420 |
| hsa-miR-569 | 0.003 | 0.018 | 0.420 |
| hsa-miR-512-5p | 0.003 | 0.018 | 0.420 |
| hsa-miR-4283 | 0.003 | 0.018 | 0.420 |
| hsa-miR-3201 | 0.003 | 0.018 | 0.420 |
| hsa-miR-4457 | 0.003 | 0.018 | 0.420 |
| hsa-miR-5693 | 0.004 | 0.018 | 0.420 |
| hsa-miR-549a | 0.004 | 0.018 | 0.420 |
| hsa-miR-223-5p | 0.003 | 0.018 | 0.420 |
| hsa-miR-4529-5p | 0.005 | 0.018 | 0.420 |
| hsa-miR-4760-5p | 0.003 | 0.018 | 0.419 |
| hsa-miR-496 | 0.003 | 0.018 | 0.419 |
| hsa-miR-6780a-3p | 0.006 | 0.020 | 0.419 |
| hsa-miR-4731-5p | 0.003 | 0.018 | 0.419 |
| hsa-miR-4704-5p | 0.003 | 0.018 | 0.419 |
| hsa-miR-660-3p | 0.003 | 0.018 | 0.419 |
| hsa-miR-3128 | 0.004 | 0.018 | 0.419 |
| hsa-miR-3914 | 0.003 | 0.018 | 0.419 |
| hsa-miR-369-3p | 0.004 | 0.018 | 0.419 |
| hsa-miR-2115-3p | 0.004 | 0.018 | 0.419 |
| hsa-miR-6866-5p | 0.002 | 0.018 | 0.419 |
| hsa-miR-5197-3p | 0.004 | 0.018 | 0.419 |
| hsa-miR-3686 | 0.003 | 0.018 | 0.419 |
| hsa-miR-3616-5p | 0.005 | 0.019 | 0.419 |
| hsa-miR-591 | 0.003 | 0.018 | 0.419 |
| hsa-miR-384 | 0.005 | 0.018 | 0.419 |
| hsa-miR-548t-5p | 0.004 | 0.018 | 0.419 |
| hsa-miR-620 | 0.003 | 0.018 | 0.419 |
| hsa-miR-4524a-3p | 0.006 | 0.020 | 0.419 |
| hsa-miR-2276-5p | 0.003 | 0.018 | 0.419 |
| hsa-miR-7706 | 0.001 | 0.018 | 0.419 |
| hsa-miR-4431 | 0.003 | 0.018 | 0.419 |
| hsa-miR-888-5p | 0.003 | 0.018 | 0.419 |
| hsa-miR-5002-5p | 0.003 | 0.018 | 0.419 |
| hsa-miR-4798-3p | 0.004 | 0.018 | 0.419 |
| hsa-miR-4537 | 0.003 | 0.018 | 0.418 |
| hsa-miR-5009-5p | 0.002 | 0.018 | 0.418 |
| hsa-miR-4794 | 0.003 | 0.018 | 0.418 |
| hsa-miR-1269a | 0.003 | 0.018 | 0.418 |
| hsa-miR-7158-3p | 0.004 | 0.018 | 0.418 |
| hsa-miR-3667-3p | 0.003 | 0.018 | 0.418 |
| hsa-miR-8065 | 0.004 | 0.018 | 0.418 |
| hsa-miR-1827 | 0.004 | 0.018 | 0.418 |
| hsa-miR-3591-5p | 0.004 | 0.018 | 0.418 |
| hsa-miR-1265 | 0.002 | 0.018 | 0.418 |
| hsa-miR-3157-3p | 0.004 | 0.018 | 0.418 |
| hsa-miR-5682 | 0.003 | 0.018 | 0.418 |
| hsa-miR-5579-5p | 0.003 | 0.018 | 0.418 |
| hsa-miR-548ax | 0.004 | 0.018 | 0.418 |
| hsa-miR-6842-5p | 0.003 | 0.018 | 0.418 |
| hsa-miR-6766-5p | 0.002 | 0.018 | 0.418 |
| hsa-miR-325 | 0.002 | 0.018 | 0.417 |
| hsa-miR-8067 | 0.004 | 0.018 | 0.417 |
| hsa-miR-1269b | 0.003 | 0.018 | 0.417 |
| hsa-miR-4495 | 0.003 | 0.018 | 0.417 |
| hsa-miR-3152-5p | 0.003 | 0.018 | 0.417 |
| hsa-miR-548l | 0.004 | 0.018 | 0.417 |
| hsa-miR-8057 | 0.002 | 0.018 | 0.417 |
| hsa-miR-4723-5p | 0.001 | 0.018 | 0.417 |
| hsa-miR-6806-3p | 0.004 | 0.018 | 0.417 |
| hsa-miR-941 | 0.003 | 0.018 | 0.417 |
| hsa-miR-4670-3p | 0.003 | 0.018 | 0.417 |
| hsa-miR-3167 | 0.003 | 0.018 | 0.417 |
| hsa-miR-6733-5p | 0.003 | 0.018 | 0.417 |
| hsa-miR-624-3p | 0.003 | 0.018 | 0.417 |
| hsa-miR-7160-3p | 0.002 | 0.018 | 0.417 |
| hsa-miR-4678 | 0.004 | 0.018 | 0.417 |
| hsa-miR-550b-3p | 0.003 | 0.018 | 0.417 |
| hsa-miR-548as-5p | 0.004 | 0.018 | 0.417 |
| hsa-miR-3683 | 0.003 | 0.018 | 0.417 |
| hsa-miR-558 | 0.003 | 0.018 | 0.416 |
| hsa-miR-3612 | 0.003 | 0.018 | 0.416 |
| hsa-miR-302d-3p | 0.003 | 0.018 | 0.416 |
| hsa-miR-219b-3p | 0.003 | 0.018 | 0.416 |
| hsa-miR-1298-5p | 0.003 | 0.018 | 0.416 |
| hsa-miR-6770-3p | 0.004 | 0.018 | 0.416 |
| hsa-miR-3908 | 0.002 | 0.018 | 0.416 |
| hsa-miR-376c-5p | 0.004 | 0.018 | 0.416 |
| hsa-miR-580-3p | 0.003 | 0.018 | 0.416 |
| hsa-miR-3140-3p | 0.003 | 0.018 | 0.415 |
| hsa-miR-3912-3p | 0.006 | 0.021 | 0.415 |
| hsa-miR-518e-3p | 0.004 | 0.018 | 0.415 |
| hsa-miR-4325 | 0.003 | 0.018 | 0.415 |
| hsa-miR-876-3p | 0.004 | 0.018 | 0.415 |
| hsa-miR-6882-3p | 0.004 | 0.018 | 0.415 |
| hsa-miR-4755-5p | 0.004 | 0.018 | 0.415 |
| hsa-miR-3134 | 0.004 | 0.018 | 0.415 |
| hsa-miR-3124-3p | 0.003 | 0.018 | 0.415 |
| hsa-miR-3942-3p | 0.004 | 0.018 | 0.415 |
| hsa-miR-124-5p | 0.003 | 0.018 | 0.415 |
| hsa-miR-2052 | 0.004 | 0.018 | 0.415 |
| hsa-miR-4500 | 0.003 | 0.018 | 0.415 |
| hsa-miR-216a-3p | 0.002 | 0.018 | 0.415 |
| hsa-miR-4690-3p | 0.003 | 0.018 | 0.415 |
| hsa-miR-3649 | 0.003 | 0.018 | 0.415 |
| hsa-miR-25-5p | 0.003 | 0.018 | 0.414 |
| hsa-miR-4633-3p | 0.003 | 0.018 | 0.414 |
| hsa-miR-3145-3p | 0.004 | 0.018 | 0.414 |
| hsa-miR-4804-5p | 0.003 | 0.018 | 0.414 |
| hsa-miR-548as-3p | 0.007 | 0.021 | 0.414 |
| hsa-miR-4438 | 0.003 | 0.018 | 0.414 |
| hsa-miR-4743-3p | 0.003 | 0.018 | 0.414 |
| hsa-miR-4490 | 0.003 | 0.018 | 0.414 |
| hsa-miR-190b | 0.004 | 0.018 | 0.414 |
| hsa-miR-4308 | 0.004 | 0.018 | 0.414 |
| hsa-miR-4797-5p | 0.003 | 0.018 | 0.414 |
| hsa-miR-3115 | 0.004 | 0.018 | 0.414 |
| hsa-miR-1283 | 0.003 | 0.018 | 0.414 |
| hsa-miR-4471 | 0.004 | 0.018 | 0.414 |
| hsa-miR-485-5p | 0.003 | 0.018 | 0.414 |
| hsa-miR-216b-3p | 0.003 | 0.018 | 0.414 |
| hsa-miR-548ad-3p | 0.004 | 0.018 | 0.413 |
| hsa-miR-4527 | 0.003 | 0.018 | 0.413 |
| hsa-miR-5009-3p | 0.004 | 0.018 | 0.413 |
| hsa-miR-4517 | 0.003 | 0.018 | 0.413 |
| hsa-miR-3133 | 0.006 | 0.020 | 0.413 |
| hsa-miR-3143 | 0.003 | 0.018 | 0.413 |
| hsa-miR-5692c | 0.002 | 0.018 | 0.413 |
| hsa-miR-5089-5p | 0.003 | 0.018 | 0.413 |
| hsa-miR-3169 | 0.003 | 0.018 | 0.413 |
| hsa-miR-1251-5p | 0.003 | 0.018 | 0.413 |
| hsa-miR-548ay-3p | 0.003 | 0.018 | 0.413 |
| hsa-miR-323b-3p | 0.003 | 0.018 | 0.413 |
| hsa-miR-4662a-3p | 0.003 | 0.018 | 0.413 |
| hsa-miR-5007-5p | 0.002 | 0.018 | 0.413 |
| hsa-miR-4803 | 0.003 | 0.018 | 0.413 |
| hsa-miR-4501 | 0.003 | 0.018 | 0.413 |
| hsa-miR-377-5p | 0.003 | 0.018 | 0.412 |
| hsa-miR-587 | 0.003 | 0.018 | 0.412 |
| hsa-miR-525-3p | 0.004 | 0.018 | 0.412 |
| hsa-miR-611 | 0.002 | 0.018 | 0.412 |
| hsa-miR-609 | 0.004 | 0.018 | 0.412 |
| hsa-miR-449c-5p | 0.003 | 0.018 | 0.412 |
| hsa-miR-6080 | 0.002 | 0.018 | 0.412 |
| hsa-miR-6883-3p | 0.004 | 0.018 | 0.412 |
| hsa-miR-548e-5p | 0.003 | 0.018 | 0.412 |
| hsa-miR-552-3p | 0.003 | 0.018 | 0.412 |
| hsa-miR-3692-3p | 0.004 | 0.018 | 0.412 |
| hsa-miR-3662 | 0.002 | 0.018 | 0.412 |
| hsa-miR-548ab | 0.005 | 0.019 | 0.411 |
| hsa-miR-4671-5p | 0.004 | 0.018 | 0.411 |
| hsa-miR-891a-3p | 0.003 | 0.018 | 0.411 |
| hsa-miR-4757-5p | 0.002 | 0.018 | 0.411 |
| hsa-miR-942-5p | 0.003 | 0.018 | 0.411 |
| hsa-miR-6840-5p | 0.002 | 0.018 | 0.411 |
| hsa-miR-4999-5p | 0.002 | 0.018 | 0.411 |
| hsa-miR-4272 | 0.003 | 0.018 | 0.411 |
| hsa-miR-4718 | 0.002 | 0.018 | 0.411 |
| hsa-miR-4802-5p | 0.003 | 0.018 | 0.411 |
| hsa-miR-3179 | 0.003 | 0.018 | 0.411 |
| hsa-miR-190a-3p | 0.004 | 0.018 | 0.411 |
| hsa-miR-105-3p | 0.003 | 0.018 | 0.411 |
| hsa-miR-3529-5p | 0.003 | 0.018 | 0.411 |
| hsa-miR-5582-3p | 0.003 | 0.018 | 0.411 |
| hsa-miR-499b-5p | 0.003 | 0.018 | 0.411 |
| hsa-miR-4795-3p | 0.005 | 0.019 | 0.411 |
| hsa-miR-548h-5p | 0.004 | 0.018 | 0.411 |
| hsa-miR-1277-3p | 0.005 | 0.019 | 0.411 |
| hsa-miR-376b-5p | 0.007 | 0.022 | 0.411 |
| hsa-miR-4477a | 0.003 | 0.018 | 0.411 |
| hsa-miR-585-3p | 0.002 | 0.018 | 0.411 |
| hsa-miR-5705 | 0.002 | 0.018 | 0.411 |
| hsa-miR-548ak | 0.005 | 0.018 | 0.411 |
| hsa-miR-520f-5p | 0.003 | 0.018 | 0.410 |
| hsa-miR-573 | 0.003 | 0.018 | 0.410 |
| hsa-miR-518e-5p | 0.003 | 0.018 | 0.410 |
| hsa-miR-203b-5p | 0.003 | 0.018 | 0.410 |
| hsa-miR-4789-3p | 0.003 | 0.018 | 0.410 |
| hsa-miR-6807-3p | 0.003 | 0.018 | 0.410 |
| hsa-miR-330-5p | 0.002 | 0.018 | 0.410 |
| hsa-miR-556-3p | 0.010 | 0.027 | 0.410 |
| hsa-miR-451b | 0.002 | 0.018 | 0.410 |
| hsa-miR-3123 | 0.003 | 0.018 | 0.410 |
| hsa-miR-6733-3p | 0.003 | 0.018 | 0.410 |
| hsa-miR-8056 | 0.004 | 0.018 | 0.410 |
| hsa-miR-920 | 0.002 | 0.018 | 0.410 |
| hsa-miR-802 | 0.005 | 0.018 | 0.410 |
| hsa-miR-621 | 0.003 | 0.018 | 0.409 |
| hsa-miR-3153 | 0.003 | 0.018 | 0.409 |
| hsa-miR-653-3p | 0.005 | 0.018 | 0.409 |
| hsa-miR-5697 | 0.003 | 0.018 | 0.409 |
| hsa-miR-520g-5p | 0.003 | 0.018 | 0.409 |
| hsa-miR-4668-3p | 0.004 | 0.018 | 0.409 |
| hsa-miR-548h-3p | 0.005 | 0.018 | 0.409 |
| hsa-miR-1245b-3p | 0.003 | 0.018 | 0.409 |
| hsa-miR-4765 | 0.004 | 0.018 | 0.408 |
| hsa-miR-1252-5p | 0.002 | 0.018 | 0.408 |
| hsa-miR-297 | 0.002 | 0.018 | 0.408 |
| hsa-miR-548ah-5p | 0.005 | 0.019 | 0.408 |
| hsa-miR-7153-5p | 0.001 | 0.018 | 0.408 |
| hsa-miR-559 | 0.002 | 0.018 | 0.408 |
| hsa-miR-4670-5p | 0.003 | 0.018 | 0.408 |
| hsa-miR-607 | 0.005 | 0.018 | 0.408 |
| hsa-miR-95-5p | 0.004 | 0.018 | 0.408 |
| hsa-miR-3657 | 0.005 | 0.019 | 0.408 |
| hsa-miR-1271-3p | 0.002 | 0.018 | 0.407 |
| hsa-miR-4759 | 0.003 | 0.018 | 0.407 |
| hsa-miR-511-5p | 0.002 | 0.018 | 0.407 |
| hsa-miR-4432 | 0.003 | 0.018 | 0.407 |
| hsa-miR-606 | 0.002 | 0.018 | 0.407 |
| hsa-miR-1302 | 0.004 | 0.018 | 0.407 |
| hsa-miR-6499-5p | 0.002 | 0.018 | 0.407 |
| hsa-miR-4503 | 0.004 | 0.018 | 0.407 |
| hsa-miR-1262 | 0.003 | 0.018 | 0.407 |
| hsa-miR-518f-3p | 0.001 | 0.018 | 0.407 |
| hsa-miR-4790-3p | 0.003 | 0.018 | 0.407 |
| hsa-miR-19a-5p | 0.003 | 0.018 | 0.407 |
| hsa-miR-1468-5p | 0.004 | 0.018 | 0.406 |
| hsa-miR-544b | 0.004 | 0.018 | 0.406 |
| hsa-miR-588 | 0.002 | 0.018 | 0.406 |
| hsa-miR-4474-5p | 0.003 | 0.018 | 0.406 |
| hsa-miR-4720-3p | 0.003 | 0.018 | 0.406 |
| hsa-miR-561-3p | 0.002 | 0.018 | 0.406 |
| hsa-miR-548at-3p | 0.003 | 0.018 | 0.406 |
| hsa-miR-302f | 0.004 | 0.018 | 0.406 |
| hsa-miR-519d-5p | 0.003 | 0.018 | 0.406 |
| hsa-miR-873-3p | 0.001 | 0.018 | 0.406 |
| hsa-miR-6854-5p | 0.002 | 0.018 | 0.406 |
| hsa-miR-759 | 0.003 | 0.018 | 0.406 |
| hsa-miR-1289 | 0.002 | 0.018 | 0.405 |
| hsa-miR-4645-5p | 0.003 | 0.018 | 0.405 |
| hsa-miR-8086 | 0.002 | 0.018 | 0.405 |
| hsa-miR-1537-3p | 0.002 | 0.018 | 0.405 |
| hsa-miR-579-3p | 0.003 | 0.018 | 0.405 |
| hsa-miR-4790-5p | 0.004 | 0.018 | 0.405 |
| hsa-miR-3117-5p | 0.002 | 0.018 | 0.405 |
| hsa-miR-3156-3p | 0.002 | 0.018 | 0.405 |
| hsa-miR-548k | 0.002 | 0.018 | 0.405 |
| hsa-miR-450b-5p | 0.003 | 0.018 | 0.405 |
| hsa-miR-3139 | 0.003 | 0.018 | 0.405 |
| hsa-miR-4662b | 0.003 | 0.018 | 0.405 |
| hsa-miR-6888-5p | 0.002 | 0.018 | 0.404 |
| hsa-miR-7154-5p | 0.002 | 0.018 | 0.404 |
| hsa-miR-4692 | 0.002 | 0.018 | 0.404 |
| hsa-miR-302b-3p | 0.002 | 0.018 | 0.404 |
| hsa-miR-200c-5p | 0.003 | 0.018 | 0.404 |
| hsa-miR-203b-3p | 0.002 | 0.018 | 0.404 |
| hsa-miR-4679 | 0.004 | 0.018 | 0.404 |
| hsa-miR-4480 | 0.003 | 0.018 | 0.404 |
| hsa-miR-4659b-5p | 0.002 | 0.018 | 0.404 |
| hsa-miR-323b-5p | 0.002 | 0.018 | 0.403 |
| hsa-miR-4659a-5p | 0.002 | 0.018 | 0.403 |
| hsa-miR-3618 | 0.002 | 0.018 | 0.403 |
| hsa-miR-4460 | 0.003 | 0.018 | 0.403 |
| hsa-miR-643 | 0.003 | 0.018 | 0.403 |
| hsa-miR-655-3p | 0.002 | 0.018 | 0.403 |
| hsa-miR-3175 | 0.003 | 0.018 | 0.403 |
| hsa-miR-3690 | 0.002 | 0.018 | 0.403 |
| hsa-miR-6755-3p | 0.007 | 0.021 | 0.403 |
| hsa-miR-876-5p | 0.003 | 0.018 | 0.403 |
| hsa-miR-4662a-5p | 0.003 | 0.018 | 0.403 |
| hsa-miR-27a-5p | 0.002 | 0.018 | 0.402 |
| hsa-miR-4680-3p | 0.002 | 0.018 | 0.402 |
| hsa-miR-3199 | 0.004 | 0.018 | 0.402 |
| hsa-miR-4652-5p | 0.003 | 0.018 | 0.402 |
| hsa-miR-2682-5p | 0.002 | 0.018 | 0.402 |
| hsa-miR-155-3p | 0.003 | 0.018 | 0.402 |
| hsa-miR-3664-5p | 0.003 | 0.018 | 0.402 |
| hsa-miR-372-3p | 0.002 | 0.018 | 0.402 |
| hsa-miR-4696 | 0.003 | 0.018 | 0.401 |
| hsa-miR-4643 | 0.004 | 0.018 | 0.401 |
| hsa-miR-4782-5p | 0.003 | 0.018 | 0.401 |
| hsa-miR-3920 | 0.003 | 0.018 | 0.401 |
| hsa-miR-5580-5p | 0.002 | 0.018 | 0.401 |
| hsa-miR-5583-3p | 0.003 | 0.018 | 0.401 |
| hsa-miR-644a | 0.002 | 0.018 | 0.401 |
| hsa-miR-8066 | 0.004 | 0.018 | 0.401 |
| hsa-miR-624-5p | 0.002 | 0.018 | 0.401 |
| hsa-miR-7159-3p | 0.003 | 0.018 | 0.401 |
| hsa-miR-767-5p | 0.001 | 0.018 | 0.401 |
| hsa-miR-4445-5p | 0.005 | 0.018 | 0.401 |
| hsa-miR-5197-5p | 0.002 | 0.018 | 0.401 |
| hsa-miR-5007-3p | 0.003 | 0.018 | 0.401 |
| hsa-miR-1273a | 0.002 | 0.018 | 0.400 |
| hsa-miR-568 | 0.002 | 0.018 | 0.400 |
| hsa-miR-6837-3p | 0.003 | 0.018 | 0.400 |
| hsa-miR-4540 | 0.001 | 0.018 | 0.400 |
| hsa-miR-548s | 0.003 | 0.018 | 0.400 |
| hsa-miR-4766-3p | 0.003 | 0.018 | 0.399 |
| hsa-miR-4666a-3p | 0.003 | 0.018 | 0.399 |
| hsa-miR-3675-5p | 0.003 | 0.018 | 0.399 |
| hsa-miR-4452 | 0.003 | 0.018 | 0.399 |
| hsa-miR-3929 | 0.002 | 0.018 | 0.399 |
| hsa-miR-2053 | 0.005 | 0.018 | 0.399 |
| hsa-miR-670-3p | 0.001 | 0.018 | 0.399 |
| hsa-miR-4509 | 0.002 | 0.018 | 0.399 |
| hsa-miR-552-5p | 0.002 | 0.018 | 0.399 |
| hsa-miR-548p | 0.002 | 0.018 | 0.399 |
| hsa-miR-208b-3p | 0.003 | 0.018 | 0.398 |
| hsa-miR-651-3p | 0.003 | 0.018 | 0.398 |
| hsa-miR-380-3p | 0.003 | 0.018 | 0.398 |
| hsa-miR-302a-3p | 0.001 | 0.018 | 0.397 |
| hsa-miR-105-5p | 0.002 | 0.018 | 0.397 |
| hsa-miR-1278 | 0.003 | 0.018 | 0.397 |
| hsa-miR-4798-5p | 0.002 | 0.018 | 0.397 |
| hsa-miR-4775 | 0.006 | 0.019 | 0.397 |
| hsa-miR-7852-3p | 0.003 | 0.018 | 0.397 |
| hsa-miR-7112-5p | 0.002 | 0.018 | 0.397 |
| hsa-miR-3672 | 0.005 | 0.019 | 0.397 |
| hsa-miR-548a-3p | 0.003 | 0.018 | 0.397 |
| hsa-miR-7156-5p | 0.002 | 0.018 | 0.397 |
| hsa-miR-553 | 0.002 | 0.018 | 0.396 |
| hsa-miR-374a-3p | 0.003 | 0.018 | 0.396 |
| hsa-miR-548ba | 0.004 | 0.018 | 0.396 |
| hsa-miR-448 | 0.004 | 0.018 | 0.396 |
| hsa-miR-4799-3p | 0.002 | 0.018 | 0.395 |
| hsa-miR-4999-3p | 0.001 | 0.018 | 0.395 |
| hsa-miR-1179 | 0.002 | 0.018 | 0.395 |
| hsa-miR-4704-3p | 0.003 | 0.018 | 0.395 |
| hsa-miR-4777-3p | 0.004 | 0.018 | 0.394 |
| hsa-miR-367-3p | 0.001 | 0.018 | 0.394 |
| hsa-miR-2114-5p | 0.002 | 0.018 | 0.393 |
| hsa-miR-3140-5p | 0.003 | 0.018 | 0.393 |
| hsa-miR-3606-3p | 0.002 | 0.018 | 0.393 |
| hsa-miR-626 | 0.002 | 0.018 | 0.393 |
| hsa-miR-548g-3p | 0.003 | 0.018 | 0.393 |
| hsa-miR-203a-5p | 0.002 | 0.018 | 0.393 |
| hsa-miR-374c-3p | 0.002 | 0.018 | 0.392 |
| hsa-miR-19b-2-5p | 0.004 | 0.018 | 0.392 |
| hsa-miR-412-5p | 0.003 | 0.018 | 0.392 |
| hsa-miR-6502-3p | 0.002 | 0.018 | 0.392 |
| hsa-miR-4473 | 0.003 | 0.018 | 0.391 |
| hsa-miR-539-3p | 0.004 | 0.018 | 0.391 |
| hsa-miR-519b-3p | 0.003 | 0.018 | 0.391 |
| hsa-miR-3670 | 0.003 | 0.018 | 0.391 |
| hsa-miR-4768-5p | 0.002 | 0.018 | 0.391 |
| hsa-miR-4789-5p | 0.002 | 0.018 | 0.391 |
| hsa-miR-4735-3p | 0.003 | 0.018 | 0.391 |
| hsa-miR-4712-5p | 0.003 | 0.018 | 0.390 |
| hsa-miR-3118 | 0.003 | 0.018 | 0.390 |
| hsa-miR-1324 | 0.003 | 0.018 | 0.390 |
| hsa-miR-329-3p | 0.003 | 0.018 | 0.389 |
| hsa-miR-515-5p | 0.002 | 0.018 | 0.389 |
| hsa-miR-3916 | 0.002 | 0.018 | 0.389 |
| hsa-miR-2909 | 0.004 | 0.018 | 0.389 |
| hsa-miR-544a | 0.003 | 0.018 | 0.389 |
| hsa-miR-518c-3p | 0.003 | 0.018 | 0.389 |
| hsa-miR-367-5p | 0.003 | 0.018 | 0.389 |
| hsa-miR-4632-3p | 0.003 | 0.018 | 0.389 |
| hsa-miR-891b | 0.004 | 0.018 | 0.388 |
| hsa-miR-6508-3p | 0.002 | 0.018 | 0.388 |
| hsa-miR-580-5p | 0.002 | 0.018 | 0.388 |
| hsa-miR-3171 | 0.004 | 0.018 | 0.388 |
| hsa-miR-3119 | 0.002 | 0.018 | 0.388 |
| hsa-miR-215-3p | 0.002 | 0.018 | 0.388 |
| hsa-miR-4757-3p | 0.001 | 0.018 | 0.387 |
| hsa-miR-374b-3p | 0.002 | 0.018 | 0.387 |
| hsa-miR-548ao-5p | 0.002 | 0.018 | 0.387 |
| hsa-miR-548m | 0.002 | 0.018 | 0.387 |
| hsa-miR-3613-5p | 0.005 | 0.019 | 0.387 |
| hsa-miR-4680-5p | 0.002 | 0.018 | 0.386 |
| hsa-miR-3661 | 0.001 | 0.018 | 0.386 |
| hsa-miR-4738-5p | 0.003 | 0.018 | 0.386 |
| hsa-miR-519c-3p | 0.005 | 0.018 | 0.386 |
| hsa-miR-5087 | 0.002 | 0.018 | 0.385 |
| hsa-miR-2355-5p | 0.001 | 0.018 | 0.385 |
| hsa-miR-1256 | 0.002 | 0.018 | 0.385 |
| hsa-miR-888-3p | 0.002 | 0.018 | 0.385 |
| hsa-miR-3912-5p | 0.002 | 0.018 | 0.384 |
| hsa-miR-1537-5p | 0.004 | 0.018 | 0.384 |
| hsa-miR-4295 | 0.002 | 0.018 | 0.384 |
| hsa-miR-3680-5p | 0.003 | 0.018 | 0.384 |
| hsa-miR-3913-5p | 0.002 | 0.018 | 0.383 |
| hsa-miR-1245b-5p | 0.002 | 0.018 | 0.383 |
| hsa-miR-3614-3p | 0.003 | 0.018 | 0.382 |
| hsa-miR-4720-5p | 0.002 | 0.018 | 0.382 |
| hsa-miR-3977 | 0.005 | 0.019 | 0.381 |
| hsa-miR-5011-3p | 0.003 | 0.018 | 0.381 |
| hsa-miR-627-3p | 0.003 | 0.018 | 0.380 |
| hsa-miR-7162-5p | 0.002 | 0.018 | 0.379 |
| hsa-miR-548am-3p | 0.002 | 0.018 | 0.379 |
| hsa-miR-5590-5p | 0.002 | 0.018 | 0.378 |
| hsa-miR-6816-5p | 0.003 | 0.018 | 0.378 |
| hsa-miR-603 | 0.002 | 0.018 | 0.376 |
| hsa-miR-548ac | 0.002 | 0.018 | 0.373 |
| hsa-miR-5579-3p | 0.003 | 0.018 | 0.371 |
| hsa-miR-301b-3p | 0.009 | 0.025 | 0.370 |
| hsa-miR-4705 | 0.001 | 0.018 | 0.370 |
| hsa-miR-633 | 0.001 | 0.018 | 0.370 |
| hsa-miR-23b-5p | 0.005 | 0.018 | 0.370 |
| hsa-miR-1252-3p | 0.003 | 0.018 | 0.368 |
| hsa-miR-8485 | 0.006 | 0.020 | 0.366 |
| hsa-miR-208b-5p | 0.005 | 0.019 | 0.365 |
| hsa-miR-26a-2-3p | 0.002 | 0.018 | 0.365 |
| hsa-miR-522-3p | 0.001 | 0.018 | 0.363 |
| hsa-miR-6844 | 0.001 | 0.018 | 0.363 |
| hsa-miR-889-3p | 0.004 | 0.018 | 0.362 |
| hsa-miR-3152-3p | 0.002 | 0.018 | 0.350 |
| hsa-miR-517a-3p | 0.001 | 0.018 | 0.344 |
| hsa-miR-503-5p | 0.003 | 0.018 | 0.329 |
| hsa-miR-517c-3p | 0.001 | 0.018 | 0.314 |
| hsa-miR-29b-1-5p | 0.001 | 0.018 | 0.307 |
| hsa-miR-455-5p | 0.009 | 0.026 | 0.294 |
| hsa-miR-424-5p | 0.006 | 0.020 | 0.292 |
| hsa-miR-24-1-5p | 0.006 | 0.020 | 0.291 |
| hsa-miR-22-5p | 0.007 | 0.022 | 0.281 |
| hsa-miR-3182 | 0.000 | 0.018 | 0.281 |
| hsa-miR-1260b | 0.001 | 0.018 | 0.263 |
| hsa-miR-1260a | 0.003 | 0.018 | 0.258 |
| hsa-miR-455-3p | 0.002 | 0.018 | 0.154 |
