# Supplementary material for: miRNA Expression Characterizes Histological Subtypes and Metastasis in Penile Squamous Cell Carcinoma
Source: Cancers (Basel). 2021 Mar 23;13(6):1480. doi: 10.3390/cancers13061480 (PMC8004785; doi:10.3390/cancers13061480)
Supplement: Supplementary file 1 [file cancers-13-01480-s001.zip › cancers-1116103-supp/Table S3.docx]

Table S3. Significantly differentially expressed miRNAs in usual (HPV-negative) PSCC versus normal samples

| Systematic name | p-value | q-value | Fold change |
| --- | --- | --- | --- |
| hsa-miR-4512 | 0.039 | 0.238 | 5.115 |
| hsa-miR-6760-5p | 0.031 | 0.238 | 3.463 |
| hsa-miR-3074-3p | 0.024 | 0.238 | 3.284 |
| hsa-miR-6850-3p | 0.010 | 0.238 | 2.784 |
| hsa-miR-4785 | 0.047 | 0.238 | 2.738 |
| hsa-miR-137 | 0.041 | 0.238 | 2.709 |
| hsa-miR-4726-3p | 0.031 | 0.238 | 2.614 |
| hsa-miR-6815-3p | 0.031 | 0.238 | 2.603 |
| **hsa-miR-105-5p** | **0.010** | **0.238** | **2.601** |
| hsa-miR-6885-5p | 0.017 | 0.238 | 2.490 |
| hsa-miR-3124-5p | 0.044 | 0.238 | 2.470 |
| hsa-miR-1343-3p | 0.041 | 0.238 | 2.420 |
| hsa-miR-522-3p | 0.048 | 0.238 | 2.401 |
| hsa-miR-5009-5p | 0.015 | 0.238 | 2.378 |
| hsa-miR-6747-3p | 0.041 | 0.238 | 2.287 |
| hsa-miR-218-2-3p | 0.031 | 0.238 | 2.275 |
| hsa-miR-6514-3p | 0.019 | 0.238 | 2.271 |
| hsa-miR-6729-3p | 0.032 | 0.238 | 2.262 |
| hsa-miR-138-5p | 0.017 | 0.238 | 2.260 |
| hsa-miR-548au-3p | 0.031 | 0.238 | 2.222 |
| hsa-miR-2115-3p | 0.045 | 0.238 | 2.220 |
| hsa-miR-888-3p | 0.036 | 0.238 | 2.215 |
| hsa-miR-5583-3p | 0.038 | 0.238 | 2.204 |
| hsa-miR-376a-2-5p | 0.042 | 0.238 | 2.202 |
| hsa-miR-4720-5p | 0.034 | 0.238 | 2.199 |
| hsa-miR-6761-3p | 0.016 | 0.238 | 2.196 |
| hsa-miR-6499-3p | 0.048 | 0.238 | 2.193 |
| hsa-miR-935 | 0.033 | 0.238 | 2.177 |
| hsa-miR-18a-3p | 0.042 | 0.238 | 2.176 |
| hsa-miR-548ac | 0.043 | 0.238 | 2.172 |
| hsa-miR-7159-3p | 0.039 | 0.238 | 2.169 |
| hsa-miR-548a-3p | 0.045 | 0.238 | 2.168 |
| hsa-miR-4680-5p | 0.038 | 0.238 | 2.165 |
| hsa-miR-4635 | 0.034 | 0.238 | 2.165 |
| hsa-miR-6816-5p | 0.043 | 0.238 | 2.162 |
| hsa-miR-6772-3p | 0.024 | 0.238 | 2.154 |
| hsa-miR-184 | 0.003 | 0.238 | 2.145 |
| hsa-miR-488-3p | 0.048 | 0.238 | 2.145 |
| hsa-miR-4786-3p | 0.028 | 0.238 | 2.130 |
| hsa-miR-942-5p | 0.032 | 0.238 | 2.122 |
| hsa-miR-185-3p | 0.040 | 0.238 | 2.117 |
| hsa-miR-563 | 0.018 | 0.238 | 2.117 |
| hsa-miR-5582-3p | 0.041 | 0.238 | 2.115 |
| hsa-miR-548ai | 0.031 | 0.238 | 2.114 |
| hsa-miR-7107-3p | 0.036 | 0.238 | 2.114 |
| hsa-miR-518e-5p | 0.030 | 0.238 | 2.110 |
| hsa-miR-4649-5p | 0.038 | 0.238 | 2.109 |
| hsa-miR-4652-3p | 0.018 | 0.238 | 2.108 |
| hsa-miR-6512-3p | 0.037 | 0.238 | 2.098 |
| hsa-miR-6502-3p | 0.045 | 0.238 | 2.092 |
| hsa-miR-6789-3p | 0.029 | 0.238 | 2.090 |
| hsa-miR-8070 | 0.043 | 0.238 | 2.083 |
| hsa-miR-367-3p | 0.042 | 0.238 | 2.078 |
| hsa-miR-548m | 0.045 | 0.238 | 2.077 |
| hsa-miR-767-5p | 0.039 | 0.238 | 2.067 |
| hsa-miR-6883-3p | 0.045 | 0.238 | 2.063 |
| hsa-miR-6505-5p | 0.047 | 0.238 | 2.063 |
| hsa-miR-433-5p | 0.043 | 0.238 | 2.060 |
| hsa-miR-4477b | 0.046 | 0.238 | 2.056 |
| hsa-miR-4540 | 0.050 | 0.238 | 2.053 |
| hsa-miR-8057 | 0.043 | 0.238 | 2.048 |
| hsa-miR-6080 | 0.046 | 0.238 | 2.047 |
| hsa-miR-520f-5p | 0.039 | 0.238 | 2.044 |
| hsa-miR-216a-3p | 0.047 | 0.238 | 2.043 |
| hsa-miR-6508-3p | 0.039 | 0.238 | 2.041 |
| hsa-miR-4705 | 0.050 | 0.238 | 2.040 |
| hsa-miR-568 | 0.036 | 0.238 | 2.040 |
| hsa-miR-450a-2-3p | 0.043 | 0.238 | 2.039 |
| hsa-miR-499b-3p | 0.046 | 0.238 | 2.034 |
| hsa-miR-6750-5p | 0.033 | 0.238 | 2.032 |
| hsa-miR-4718 | 0.045 | 0.238 | 2.031 |
| hsa-miR-526b-5p | 0.024 | 0.238 | 2.031 |
| hsa-miR-518d-3p | 0.048 | 0.238 | 2.028 |
| hsa-miR-4653-5p | 0.043 | 0.238 | 2.027 |
| hsa-miR-4666a-5p | 0.038 | 0.238 | 2.021 |
| hsa-miR-8086 | 0.047 | 0.238 | 2.019 |
| hsa-miR-92a-2-5p | 0.044 | 0.238 | 2.017 |
| hsa-miR-6844 | 0.045 | 0.238 | 2.017 |
| hsa-miR-561-5p | 0.038 | 0.238 | 2.016 |
| hsa-miR-943 | 0.040 | 0.238 | 2.016 |
| hsa-miR-4640-3p | 0.039 | 0.238 | 2.015 |
| hsa-miR-2276-5p | 0.049 | 0.238 | 2.014 |
| hsa-miR-668-5p | 0.041 | 0.238 | 2.010 |
| hsa-miR-1245b-5p | 0.043 | 0.238 | 2.009 |
| hsa-miR-6883-5p | 0.047 | 0.238 | 2.009 |
| hsa-miR-7153-5p | 0.035 | 0.238 | 2.009 |
| hsa-miR-8083 | 0.046 | 0.238 | 2.008 |
| hsa-miR-920 | 0.046 | 0.238 | 2.007 |
| hsa-miR-3674 | 0.024 | 0.238 | 2.004 |
| hsa-miR-29b-2-5p | 0.045 | 0.238 | 2.003 |
| hsa-miR-6759-3p | 0.048 | 0.238 | 2.002 |
| hsa-miR-548b-5p | 0.050 | 0.238 | 1.999 |
| hsa-miR-6835-3p | 0.049 | 0.238 | 1.997 |
| hsa-miR-520d-3p | 0.033 | 0.238 | 1.996 |
| hsa-miR-4310 | 0.034 | 0.238 | 1.995 |
| hsa-miR-6804-3p | 0.037 | 0.238 | 1.995 |
| hsa-miR-6888-5p | 0.045 | 0.238 | 1.995 |
| hsa-miR-6768-3p | 0.038 | 0.238 | 1.995 |
| hsa-miR-6767-3p | 0.047 | 0.238 | 1.993 |
| hsa-miR-1204 | 0.041 | 0.238 | 1.990 |
| hsa-miR-4999-3p | 0.048 | 0.238 | 1.987 |
| hsa-miR-4265 | 0.036 | 0.238 | 1.984 |
| hsa-miR-4761-3p | 0.047 | 0.238 | 1.982 |
| hsa-miR-6776-3p | 0.039 | 0.238 | 1.978 |
| hsa-miR-584-3p | 0.039 | 0.238 | 1.976 |
| hsa-miR-559 | 0.046 | 0.238 | 1.971 |
| hsa-miR-4659a-5p | 0.045 | 0.238 | 1.971 |
| hsa-miR-6891-3p | 0.040 | 0.238 | 1.971 |
| hsa-miR-6749-3p | 0.039 | 0.238 | 1.970 |
| hsa-miR-1304-3p | 0.034 | 0.238 | 1.970 |
| hsa-miR-3151-5p | 0.030 | 0.238 | 1.969 |
| hsa-miR-2116-3p | 0.031 | 0.238 | 1.969 |
| hsa-miR-6728-3p | 0.023 | 0.238 | 1.968 |
| hsa-miR-5699-3p | 0.043 | 0.238 | 1.968 |
| hsa-miR-1271-3p | 0.048 | 0.238 | 1.965 |
| hsa-miR-626 | 0.048 | 0.238 | 1.964 |
| hsa-miR-3529-3p | 0.048 | 0.238 | 1.961 |
| hsa-miR-7158-5p | 0.036 | 0.238 | 1.960 |
| hsa-miR-4723-5p | 0.043 | 0.238 | 1.960 |
| hsa-miR-708-3p | 0.050 | 0.238 | 1.958 |
| hsa-miR-1825 | 0.046 | 0.238 | 1.957 |
| hsa-miR-6838-5p | 0.043 | 0.238 | 1.952 |
| hsa-miR-6818-5p | 0.044 | 0.238 | 1.951 |
| hsa-miR-1253 | 0.045 | 0.238 | 1.951 |
| hsa-miR-567 | 0.040 | 0.238 | 1.950 |
| hsa-miR-7152-5p | 0.021 | 0.238 | 1.946 |
| hsa-miR-4692 | 0.049 | 0.238 | 1.942 |
| hsa-miR-6732-3p | 0.024 | 0.238 | 1.937 |
| hsa-miR-6851-3p | 0.035 | 0.238 | 1.934 |
| hsa-miR-23c | 0.029 | 0.238 | 1.931 |
| hsa-miR-106b-3p | 0.046 | 0.238 | 1.927 |
| hsa-miR-4666b | 0.032 | 0.238 | 1.923 |
| hsa-miR-5006-3p | 0.028 | 0.238 | 1.920 |
| hsa-miR-7706 | 0.038 | 0.238 | 1.915 |
| hsa-miR-6773-5p | 0.047 | 0.238 | 1.905 |
| hsa-miR-593-3p | 0.050 | 0.238 | 1.903 |
| hsa-miR-1468-3p | 0.047 | 0.238 | 1.902 |
| hsa-miR-8054 | 0.042 | 0.238 | 1.897 |
| hsa-miR-6865-3p | 0.049 | 0.238 | 1.893 |
| hsa-miR-6737-3p | 0.039 | 0.238 | 1.892 |
| hsa-miR-4300 | 0.039 | 0.238 | 1.882 |
| hsa-let-7f-1-3p | 0.049 | 0.238 | 1.881 |
| hsa-miR-658 | 0.046 | 0.238 | 1.880 |
| hsa-miR-6515-5p | 0.040 | 0.238 | 1.872 |
| hsa-miR-1227-3p | 0.047 | 0.238 | 1.871 |
| hsa-miR-4447 | 0.008 | 0.238 | 1.870 |
| hsa-miR-4737 | 0.039 | 0.238 | 1.861 |
| hsa-miR-6735-5p | 0.046 | 0.238 | 1.857 |
| hsa-miR-6858-3p | 0.044 | 0.238 | 1.856 |
| hsa-miR-3613-3p | 0.039 | 0.238 | 1.852 |
| hsa-miR-6861-3p | 0.040 | 0.238 | 1.851 |
| hsa-miR-6825-5p | 0.015 | 0.238 | 1.839 |
| hsa-miR-7161-3p | 0.049 | 0.238 | 1.831 |
| hsa-miR-7114-3p | 0.041 | 0.238 | 1.814 |
| hsa-miR-6855-3p | 0.034 | 0.238 | 1.812 |
| hsa-miR-4319 | 0.034 | 0.238 | 1.805 |
| hsa-miR-4700-5p | 0.036 | 0.238 | 1.787 |
| hsa-miR-5571-5p | 0.039 | 0.238 | 1.785 |
| hsa-miR-518c-5p | 0.049 | 0.238 | 1.782 |
| hsa-miR-6857-3p | 0.033 | 0.238 | 1.780 |
| hsa-miR-1266-5p | 0.044 | 0.238 | 1.737 |
| hsa-miR-7113-5p | 0.045 | 0.238 | 1.731 |
| hsa-miR-6503-3p | 0.021 | 0.238 | 1.722 |
| hsa-miR-7108-3p | 0.045 | 0.238 | 1.700 |
| hsa-miR-2355-3p | 0.032 | 0.238 | 1.697 |
| hsa-miR-3180-5p | 0.015 | 0.238 | 1.692 |
| hsa-miR-4682 | 0.028 | 0.238 | 1.679 |
| hsa-miR-4748 | 0.035 | 0.238 | 1.678 |
| hsa-miR-3680-3p | 0.005 | 0.238 | 1.664 |
| hsa-miR-4676-5p | 0.049 | 0.238 | 1.639 |
| hsa-miR-6718-5p | 0.036 | 0.238 | 1.616 |
| hsa-miR-6802-5p | 0.019 | 0.238 | 1.582 |
| hsa-miR-4667-3p | 0.046 | 0.238 | 1.581 |
| hsa-miR-625-3p | 0.043 | 0.238 | 1.566 |
| hsa-miR-6790-3p | 0.026 | 0.238 | 1.378 |
| hsa-miR-370-3p | 0.049 | 0.238 | 0.504 |
| hsa-miR-489-3p | 0.031 | 0.238 | 0.496 |
| **hsa-miR-125b-2-3p** | **0.018** | **0.238** | **0.424** |
| hsa-miR-17-3p | 0.029 | 0.238 | 0.410 |
| **hsa-miR-99a-5p** | **0.037** | **0.238** | **0.124** |
