# Supplementary material for: miRNA Expression Characterizes Histological Subtypes and Metastasis in Penile Squamous Cell Carcinoma
Source: Cancers (Basel). 2021 Mar 23;13(6):1480. doi: 10.3390/cancers13061480 (PMC8004785; doi:10.3390/cancers13061480)
Supplement: Supplementary file 1 [file cancers-13-01480-s001.zip › cancers-1116103-supp/Table S4.docx]

Table S4. Significantly differentially expressed miRNAs in metastatic versus non-metastatic usual (HPV-negative) PSCC.

| Systematic name | p-value | q-value | Fold change |
| --- | --- | --- | --- |
| hsa-miR-4498 | 0.001 | 0.082 | 5.466 |
| hsa-miR-3692-5p | 0.001 | 0.082 | 4.813 |
| hsa-miR-513c-5p | 0.006 | 0.082 | 4.251 |
| **hsa-miR-509-5p** | **0.005** | **0.082** | **3.825** |
| hsa-miR-6132 | 0.001 | 0.082 | 3.669 |
| hsa-miR-3666 | 0.001 | 0.082 | 3.479 |
| hsa-miR-4733-5p | 0.001 | 0.082 | 3.441 |
| hsa-miR-4647 | 0.002 | 0.082 | 3.402 |
| hsa-miR-6847-5p | 0.001 | 0.082 | 3.386 |
| hsa-miR-6798-5p | 0.001 | 0.082 | 3.379 |
| hsa-miR-6857-5p | 0.001 | 0.082 | 3.342 |
| hsa-miR-9500 | 0.000 | 0.082 | 3.180 |
| hsa-miR-3197 | 0.001 | 0.082 | 2.993 |
| hsa-miR-4462 | 0.001 | 0.082 | 2.927 |
| hsa-miR-370-3p | 0.000 | 0.082 | 2.892 |
| hsa-miR-10b-3p | 0.006 | 0.082 | 2.872 |
| hsa-miR-512-3p | 0.000 | 0.082 | 2.727 |
| hsa-miR-3130-5p | 0.009 | 0.082 | 2.671 |
| hsa-miR-6830-5p | 0.007 | 0.082 | 2.606 |
| hsa-miR-921 | 0.002 | 0.082 | 2.598 |
| hsa-miR-1273e | 0.004 | 0.082 | 2.571 |
| hsa-miR-4468 | 0.005 | 0.082 | 2.533 |
| hsa-miR-1208 | 0.002 | 0.082 | 2.431 |
| hsa-miR-662 | 0.005 | 0.082 | 2.395 |
| hsa-miR-617 | 0.005 | 0.082 | 2.373 |
| hsa-miR-4673 | 0.009 | 0.082 | 2.249 |
| hsa-miR-8085 | 0.003 | 0.082 | 2.164 |
| hsa-miR-4294 | 0.009 | 0.082 | 2.112 |
| hsa-miR-331-5p | 0.006 | 0.082 | 0.496 |
| hsa-miR-1229-5p | 0.007 | 0.082 | 0.496 |
| hsa-miR-129-5p | 0.002 | 0.082 | 0.496 |
| hsa-miR-6749-5p | 0.003 | 0.082 | 0.496 |
| hsa-miR-6723-5p | 0.008 | 0.082 | 0.491 |
| hsa-miR-6890-3p | 0.006 | 0.082 | 0.478 |
| hsa-miR-6880-3p | 0.005 | 0.082 | 0.477 |
| hsa-miR-4303 | 0.008 | 0.082 | 0.476 |
| hsa-miR-6812-3p | 0.003 | 0.082 | 0.471 |
| hsa-miR-766-3p | 0.001 | 0.082 | 0.470 |
| hsa-miR-92b-3p | 0.009 | 0.082 | 0.469 |
| hsa-miR-6760-3p | 0.008 | 0.082 | 0.464 |
| hsa-miR-3155a | 0.006 | 0.082 | 0.461 |
| hsa-miR-7114-3p | 0.005 | 0.082 | 0.458 |
| hsa-miR-4728-3p | 0.002 | 0.082 | 0.458 |
| hsa-miR-30d-3p | 0.007 | 0.082 | 0.454 |
| hsa-miR-6819-3p | 0.005 | 0.082 | 0.453 |
| hsa-miR-6732-3p | 0.003 | 0.082 | 0.450 |
| hsa-miR-6813-3p | 0.007 | 0.082 | 0.450 |
| hsa-miR-3181 | 0.009 | 0.082 | 0.450 |
| hsa-miR-4769-3p | 0.002 | 0.082 | 0.448 |
| hsa-miR-629-5p | 0.001 | 0.082 | 0.445 |
| hsa-miR-627-5p | 0.008 | 0.082 | 0.445 |
| hsa-miR-4801 | 0.009 | 0.082 | 0.444 |
| hsa-miR-6861-3p | 0.003 | 0.082 | 0.444 |
| hsa-miR-2114-3p | 0.006 | 0.082 | 0.442 |
| hsa-miR-6772-3p | 0.009 | 0.082 | 0.441 |
| hsa-miR-4436b-5p | 0.003 | 0.082 | 0.440 |
| hsa-miR-6834-3p | 0.009 | 0.082 | 0.439 |
| hsa-miR-4763-5p | 0.010 | 0.082 | 0.439 |
| hsa-miR-6073 | 0.002 | 0.082 | 0.436 |
| hsa-miR-1301-3p | 0.005 | 0.082 | 0.435 |
| hsa-miR-6865-3p | 0.007 | 0.082 | 0.434 |
| hsa-miR-3162-3p | 0.006 | 0.082 | 0.432 |
| hsa-miR-3940-3p | 0.004 | 0.082 | 0.431 |
| hsa-miR-1243 | 0.007 | 0.082 | 0.427 |
| hsa-miR-4723-3p | 0.008 | 0.082 | 0.427 |
| hsa-miR-6874-5p | 0.002 | 0.082 | 0.426 |
| hsa-miR-939-3p | 0.005 | 0.082 | 0.425 |
| hsa-miR-7160-3p | 0.008 | 0.082 | 0.422 |
| hsa-miR-106b-3p | 0.006 | 0.082 | 0.421 |
| hsa-miR-6871-3p | 0.008 | 0.082 | 0.420 |
| hsa-miR-1296-5p | 0.001 | 0.082 | 0.420 |
| hsa-miR-138-1-3p | 0.001 | 0.082 | 0.417 |
| hsa-miR-1204 | 0.007 | 0.082 | 0.417 |
| hsa-miR-5000-5p | 0.002 | 0.082 | 0.416 |
| hsa-miR-1233-3p | 0.009 | 0.082 | 0.416 |
| hsa-miR-7161-3p | 0.002 | 0.082 | 0.414 |
| hsa-miR-545-3p | 0.003 | 0.082 | 0.414 |
| hsa-miR-33a-3p | 0.009 | 0.082 | 0.413 |
| hsa-miR-6875-3p | 0.002 | 0.082 | 0.411 |
| hsa-miR-624-5p | 0.008 | 0.082 | 0.408 |
| hsa-miR-8485 | 0.007 | 0.082 | 0.408 |
| hsa-miR-1825 | 0.005 | 0.082 | 0.404 |
| hsa-miR-5682 | 0.009 | 0.082 | 0.403 |
| hsa-miR-6823-3p | 0.008 | 0.082 | 0.401 |
| hsa-miR-374b-3p | 0.006 | 0.082 | 0.400 |
| hsa-miR-4664-5p | 0.006 | 0.082 | 0.399 |
| hsa-miR-5008-3p | 0.001 | 0.082 | 0.398 |
| hsa-miR-105-5p | 0.005 | 0.082 | 0.398 |
| hsa-miR-1296-3p | 0.003 | 0.082 | 0.396 |
| hsa-miR-590-3p | 0.008 | 0.082 | 0.395 |
| hsa-miR-4509 | 0.009 | 0.082 | 0.394 |
| hsa-miR-6749-3p | 0.002 | 0.082 | 0.393 |
| hsa-miR-582-3p | 0.008 | 0.082 | 0.393 |
| hsa-miR-6730-3p | 0.002 | 0.082 | 0.392 |
| hsa-miR-767-5p | 0.004 | 0.082 | 0.392 |
| hsa-miR-642a-5p | 0.010 | 0.082 | 0.387 |
| hsa-miR-93-3p | 0.004 | 0.082 | 0.387 |
| hsa-miR-2116-5p | 0.007 | 0.082 | 0.387 |
| hsa-miR-6750-3p | 0.007 | 0.082 | 0.384 |
| hsa-miR-4738-5p | 0.010 | 0.082 | 0.384 |
| hsa-miR-5704 | 0.007 | 0.082 | 0.382 |
| hsa-miR-483-3p | 0.001 | 0.082 | 0.381 |
| hsa-miR-4641 | 0.008 | 0.082 | 0.376 |
| hsa-miR-466 | 0.002 | 0.082 | 0.373 |
| hsa-miR-4321 | 0.002 | 0.082 | 0.373 |
| hsa-miR-3192-3p | 0.000 | 0.082 | 0.367 |
| hsa-miR-520g-5p | 0.007 | 0.082 | 0.366 |
| **hsa-miR-328-3p** | **0.007** | **0.082** | **0.359** |
| hsa-miR-1908-3p | 0.002 | 0.082 | 0.358 |
| hsa-miR-6735-3p | 0.005 | 0.082 | 0.356 |
| hsa-miR-2053 | 0.008 | 0.082 | 0.354 |
| hsa-miR-4634 | 0.001 | 0.082 | 0.332 |
| hsa-miR-675-3p | 0.005 | 0.082 | 0.329 |
| hsa-miR-130b-5p | 0.001 | 0.082 | 0.317 |
| hsa-miR-375 | 0.001 | 0.082 | 0.312 |
| hsa-miR-8069 | 0.008 | 0.082 | 0.298 |
| **hsa-miR-137** | **0.004** | **0.082** | **0.269** |
| hsa-miR-301b-3p | 0.007 | 0.082 | 0.246 |
